# Effects of atlas-based anatomy on modelled light transport in the neonatal head

**DOI:** 10.1088/1361-6560/acd48c

**Published:** 2023-07-03

**Authors:** Pauliina Hirvi, Topi Kuutela, Qianqian Fang, Antti Hannukainen, Nuutti Hyvönen, Ilkka Nissilä

**Affiliations:** 1 Aalto University, Department of Mathematics and Systems Analysis, PO Box 11100, FI-00076 AALTO, Finland; 2 Northeastern University, Department of Bioengineering, 360 Huntington Ave, Boston, MA 02115, United States of America; 3 Aalto University, Department of Neuroscience and Biomedical Engineering, PO Box 12200, FI-00076 AALTO, Finland

**Keywords:** approximation error, atlas models, cerebrospinal fluid, diffusion approximation, frequency-domain diffuse optical tomography, Monte Carlo methods, segmentation

## Abstract

*Objective.* Diffuse optical tomography (DOT) provides a relatively convenient method for imaging haemodynamic changes related to neuronal activity on the cerebral cortex. Due to practical challenges in obtaining anatomical images of neonates, an anatomical framework is often created from an age-appropriate atlas model, which is individualized to the subject based on measurements of the head geometry. This work studies the approximation error arising from using an atlas instead of the neonate's own anatomical model. *Approach.* We consider numerical simulations of frequency-domain (FD) DOT using two approaches, Monte Carlo simulations and diffusion approximation via finite element method, and observe the variation in (1) the logarithm of amplitude and phase shift measurements, and (2) the corresponding inner head sensitivities (Jacobians), due to varying segmented anatomy. Varying segmentations are sampled by registering 165 atlas models from a neonatal database to the head geometry of one individual selected as the reference model. Prior to the registration, we refine the segmentation of the cerebrospinal fluid (CSF) by separating the CSF into two physiologically plausible layers. *Main results.* In absolute measurements, a considerable change in the grey matter or extracerebral tissue absorption coefficient was found detectable over the anatomical variation. In difference measurements, a small local 10%-increase in brain absorption was clearly detectable in the simulated measurements over the approximation error in the Jacobians, despite the wide range of brain maturation among the registered models. *Significance.* Individual-level atlas models could potentially be selected within several weeks in gestational age in DOT difference imaging, if an exactly age-appropriate atlas is not available. The approximation error method could potentially be implemented to improve the accuracy of atlas-based imaging. The presented CSF segmentation algorithm could be useful also in other model-based imaging modalities. The computation of FD Jacobians is now available in the widely-used Monte Carlo eXtreme software.

## 1. Introduction

Diffuse optical tomography (DOT) is an emerging functional neuroimaging method suitable for mapping cortical brain activity. Multi-wavelength DOT can reconstruct the oxygenated (HbO_2_), deoxygenated (HbR) and total (HbT) arterial haemoglobin concentration changes from measured changes in the intensity of near-infrared light that has propagated through the tissue (Bluestone *et al* 2001, Nissilä *et al* 2005b). Synaptic activity modulates the arterial diameter, blood flow and volume through the neurovascular coupling, and changes in the metabolic rate of oxygen affect the relative haemoglobin concentrations (Hillman *et al* 2007). The combined vascular and metabolic effects result in the measured haemodynamic response.

The three types of DOT instrumentation include continuous-wave (CW), time-domain (TD) and frequency-domain (FD) systems (Nissilä *et al* 2005b). TD and FD measurements can be used for estimating the tissue-specific absorption and scattering coefficients, since they provide time-resolved information for separating the absorption and scattering effects (Arridge and Lionheart 1998). In this study, we model a FD system, where input light is intensity-modulated at radio-frequency (100 MHz) and the log-amplitude and phase shift of the modulation in the detected signal, or photon density wave, can be separated (Nissilä *et al* 2005a).

The portable and silent instrumentation, low environmental requirements and manageable sensitivity to movement make DOT a potential method for neurodevelopmental studies of both sleeping and awake neonates and toddlers. The child can be imaged while laying or sitting on their parent’s lap, which enables a natural environment for studying, for example, emotional speech (Shekhar *et al* 2019, Maria *et al* 2020), affective touch (Jönsson *et al* 2018, Maria *et al* 2022) and other forms of social interaction. Wireless instruments will further increase the practicality of DOT. Another important application in neonates is the detection of cerebral vasculature injuries, especially in preterm neonates in intensive care, at bedside without contrast agents (Hintz *et al* 1999, Gibson *et al* 2005). In neonates, the smaller head and thinner extracerebral layers also allow the light to propagate deeper into the brain, increasing the relative sensitivity of the measurements to the cortical tissue (Brigadoi *et al* 2014).

The DOT image reconstruction is based on the selection of a forward solver, which for a given target model, optical properties and optode (sources and detectors) locations estimates the measurements at the detectors (Arridge and Hebden 1997). In this study, we consider the two most common forward solvers: stochastic Monte Carlo (MC) simulations in voxelated models, and solving the deterministic diffusion approximation (DA) model with the finite element method (FEM) in tetrahedral mesh models. The forward solution—specifically the trajectories of the detected photons in MC and the fluence distribution in the medium in DA—is used to build the sensitivity profiles, or Jacobians, which contain the linearized relationship between the changes in the measurements and the changes in the optical properties of the medium. The linear model can be inverted to reconstruct the optical parameter changes from the measured difference data, and the parameter changes are convertible to haemoglobin concentration changes for physiological interpretation (Bluestone *et al* 2001, Gibson *et al* 2005, Maria *et al* 2022). The log-amplitude and phase shift Jacobians have different depth sensitivity profiles, which can improve the imaging accuracy (Binzoni *et al* 2017, Doulgerakis *et al* 2019).

Due to practical challenges in obtaining anatomical images of children (i.e. the required target model), an anatomical framework for functional neuroimaging studies is often created using an age-appropriate atlas model which can be individualized to the subject based on relatively simple measurements of the geometry of the outer surface of the scalp. For example, a digitizing pen or photogrammetry (Maria *et al* 2022) can be used to reconstruct a cloud of cranial landmarks, to which the geometry of the atlas model is registered. Further benefits of atlas-based modelling include that (1) only the atlas model has to be segmented, which can be a challenging task especially in neonates (Brigadoi *et al* 2014) and often requires some manual work, (2) the anatomical frame for group-level statistical analysis of the haemodynamic responses is readily available by inverse-registering the subject-specific reconstructions back to the common atlas space, and (3) the experiments are less expensive and pose fewer requirements for the instrumentation and measurement environment when anatomical images are not acquired. The atlas model can present either a population-level average or the specific anatomy of an individual in the target age group. In this study, we consider the individual-level atlas, since the averaging may result in unrealistically smooth brain features (Collins-Jones *et al* 2021). Nevertheless, another individual’s anatomy will not match the subject’s anatomy. It is unclear how the residual inaccuracy of the internal anatomy in the individualized atlas models affects the propagation and distribution of light within tissue, which motivates the current study.

Wu *et al* (2014, 2015) compared the errors in the atlas-based sensitivity, or Jacobian matrices on the brain cortex in adults for different registration algorithms (Wu *et al* 2014, 2015). Previous works have also shown the feasibility of atlas-based models in reconstructing cortical brain activity in adults (Custo *et al* 2010, Cooper *et al* 2012, Ferradal *et al* 2014, Wu *et al* 2014). Heiskala *et al* (2007, 2009b) demonstrated via simulations that atlas-based models improve the DOT imaging accuracy compared to simple homogeneous or layered models in neonates (Heiskala *et al* 2007, 2009b). Generally, the usage of atlas models in neonates requires more consideration due to the rapid growth and maturation during the last trimester of the pregnancy. Accurate age-matching is recommended (Brigadoi *et al* 2014, Collins-Jones *et al* 2021); for example, Collins-Jones *et al* (2021) use a maximum gap of ±1 week in combined gestational and chronological age when selecting the individual atlas from their public database (presented in Collins-Jones *et al* 2021; available at https://www.ucl.ac.uk/dot-hub). In the current study, we use 166 late preterm to post-term neonates from the same database by Collins-Jones *et al*, and study the expected error caused by using individual-level atlases over a larger age span of 34–43 weeks.

We select one term neonate as the reference subject, and individualize the remaining 165 atlas models for the reference neonate so that all models have exactly the same exterior head geometry and optode locations, but differing segmented inner anatomy. The segmentation includes a combined scalp and skull (S&S) layer, grey matter (GM), white matter (WM) and cerebrospinal fluid (CSF), which for MC simulations is separated into two compartments—CSF in the subarachnoid layer, and CSF in the sulci and ventricles as in Maria *et al* (2022)—with a novel algorithm. The optode locations are set by projecting our high-density measurement probe with 15 sources and detectors (Maria *et al* 2022) to the left fronto-temporal hemisphere. We simulate the measurement estimates and the Jacobians for all 165 + 1 head models, and observe the atlas modelling related approximation error in the absolute measurements and the linear approximations for the difference in the measurements due to a local HbT increase.

The magnitude of the approximation error in the absolute measurements is compared to the effect of changing the baseline absorption coefficient of the whole S&S or GM tissue type. The idea behind this is to observe the suitability of atlas models in providing measurement estimates that can be fitted to real measurements for estimating the baseline optical parameters as in Maria *et al* (2022). Baseline parameter fitting is well-motivated since values reported in literature vary between sources (Farina *et al* 2015) and parameter values differ with age, hair and skin color. The magnitude of the approximation error in the linearized difference measurements is compared to the actual simulated difference signal from the reference neonate to gain insight on the usability of individual atlases in DOT difference imaging of neuronal activity in the neonatal brain.

Our hypothesis is that the effects of the variation in the inner segmented anatomy may be amplified due to the thinness of the S&S and the proximity of the brain to the head surface in neonates. Small local or tissue-specific optical changes in the reference model may have a limited effect compared to the anatomical variation. We aim to address the suitability of the approximation error method (Arridge *et al* 2006) for modelling the anatomical inaccuracy as an additional source of noise in DOT. This could improve the imaging accuracy in situations when it is not possible or convenient to move the newborn to an anatomical scanner.

## 2. Theory and methods

### 2.1. Forward model

The forward problem in optical imaging is to determine the photon density, or fluence, when the properties of all sources and detectors as well as the optical parameters and geometry of the imaged target are provided. The radiative transfer equation (RTE) is usually considered a sufficiently accurate mathematical description for the forward problem, even though Maxwell’s equations constitute, in principle, a more accurate physical model for the light propagation in tissue. Since it is very challenging to solve the RTE in complex three-dimensional (3D) domains such as the human head, the forward solutions are usually approximated with Monte Carlo (MC) methods or by replacing the RTE with its diffusion approximation (DA) (Arridge and Hebden 1997, Nissilä *et al* 2005b). MC methods are considered to be the gold standard in accuracy of implementing the RTE (Fang and Boas 2009b). However, the MC based methods depend on the number of detected photons, making them prone to errors related to a low signal–to–noise ratio (SNR). The MC methods typically also require longer computation times, but the technical development of graphical processing units (GPU) for massively parallel simulations has significantly increased the practicality of the approach. In our case, one forward simulation (one source) with 1 × 10^9^ photons is obtained in approximately 2 minutes, versus approximately 20 seconds with the DA.

Regardless of the chosen approximation, some properties of our forward model remain the same. A head is modelled as a bounded domain ${\mathrm{\Omega }}\subset {{\mathbb{R}}}^{3}$ with a Lipschitz boundary ∂Ω and a connected complement. To facilitate numerical solution, Ω is discretized into voxels for the MC method and into a tetrahedral mesh for the DA. The employed discretization has a slight effect on the shape of ∂Ω and the segmentation of Ω into different tissue types. The photon sources and detectors are modelled as circular patches of radius *R* = 1.5 mm on ∂Ω, close to the radii in our recent experimental setups (Maria *et al* 2020, 2022). However, the exact definitions for the source and detector models differ slightly between the two forward models, and they are separately defined in the following two subsections.

The optical properties of Ω are characterized by four quantities modelled as positive integrable functions, i.e. as elements of ${L}_{+}^{\infty }({\mathrm{\Omega }})=\left\{v\in {L}^{\infty }({\mathrm{\Omega }})\,|\,{\mathrm{e}}{\mathrm{s}}{\mathrm{s}}\,{\mathrm{i}}{\mathrm{n}}{\mathrm{f}}\,v\gt 0\right\}$. They are the absorption coefficient *μ*_*a*_, the scattering coefficient *μ*_*s*_, the anisotropy coefficient *g*, and the refractive index *n*. The domain Ω is segmented into five tissue types with constant optical properties: scalp and skull (S&S), two types of cerebrospinal fluid (CSF; see section 3.2), grey matter (GM), and white matter (WM). The coefficients *μ*_*a*_ and *μ*_*s*_ are thus modelled as piece-wise constant functions obeying the segmentation of the head (and its discretization), while *n* and *g* are assumed to be constant in Ω. This also means that the speed of light *c* is modelled as constant in Ω.

#### 2.1.1. Monte Carlo simulations

The Monte Carlo forward model is a stochastic solver for the RTE based on simulating the trajectories of a large number (1 × 10^9^ in this study) of photon packets to achieve stable approximation for the photon fluence in Ω. Monte Carlo eXtreme (MCX) (Fang and Boas 2009b) (https://mcx.space) is an open-source, GPU-accelerated simulation suite that provides tools to model light transport inside voxelated (MCX), as well as mesh-based (MMC; Fang 2010) and hybrid (SVMC; Yan and Fang 2020) domain models. In this study, we use MCXLAB, which is the MATLAB (MathWorks, Natick, MA, USA) interfaced MCX for voxelated models. We utilize the detected photons’ tissue-wise partial-path-lengths (Boas *et al* 2002) collected during the MC simulation to compute statistical estimates for the ‘complex intensity’ of the frequency-modulated detected photon density waves.

We model each MCX photon source as a collimated beam originating from a disk of radius *R*, uniformly launching photon packets in the direction of the center of curvature for the measurement probe placed on the head (see section 3.3). Specular reflection that occurs due to the air between the source and the domain is not considered. The photon transport process was originally described in Fang and Boas (2009b), but has since been updated to precise ray-tracing (Fang 2010, Fang and Yan 2022). MCX implements a microscopic Beer–Lambert law (mBLL) strategy (Sassaroli and Martelli 2012), unlike the albedo-weight (AW) strategy used in some other MC codes such as MCML (Wang *et al* 1995). A benefit of using mBLL is that the photon trajectories are independent of the absorption coefficients throughout the domain, thus the absorption coefficients can be fixed (and perturbed) afterwards (Boas *et al* 2002, Yao *et al* 2018). At each scattering event within the domain, the scattering direction and length are sampled using the current voxel’s scattering and anisotropy coefficient (Wang *et al* 1995, Boas *et al* 2002, Fang and Boas 2009b). Changes in the scattering coefficient along the path are accounted for by scaling the remaining length with the ratio of the initial to the new scattering coefficient (Boas *et al* 2002). At the exterior boundary, a photon either exits or is reflected back (binary decision) according to Fresnel’s law with the reflection direction handled as described in Fang and Boas (2009b). If the photon packet exits the domain at a detector patch, it is detected and the detector index and the collected tissue-wise path-lengths for the photon in question are saved. Essentially, the photon transport is handled so that all information required for the following steps is provided by the number of detected photons and the trajectories of the photons.

Denoting by *l*_*p*,*j*_ the length of the intersection of voxel *j* and the path of a photon packet *p* exiting through one of the detectors, the weight of the photon detected at that sensor can be given as (Heiskala *et al* 2007, 2009a, Heiskala 2009)

\begin{eqnarray*}{w}_{p}=\exp \left(-\displaystyle \sum _{j}{\mu }_{a,j}\,{l}_{p,j}\right),\end{eqnarray*}

assuming the initial weight of 1. Here, *μ*_*a*,*j*_ is the constant absorption coefficient in voxel *j*. The final weight describes the probability of the corresponding real detection event, and the summed probability over all detected photons at the sensor in question approximates the relative intensity measured by the corresponding source–detector pair in comparison to other pairs. The real and imaginary correspondents of the measured intensity at the sensor are computed as (Heiskala *et al* 2007, Heiskala 2009)

\begin{eqnarray*}X=\displaystyle \sum _{p}{w}_{p}\cos (2\pi {{ft}}_{p})\quad \mathrm{and}\quad Y=\displaystyle \sum _{p}{w}_{p}\sin (2\pi {{ft}}_{p}),\end{eqnarray*}

respectively, and divided with the number of input photons for unit source strength. Here, *f* is the intensity modulation frequency of the input photon beam, and *t*_*p*_ is the total time-of-flight from launch to detection for the photon packet *p*. Based on these values, the log-amplitude $\mathrm{ln}(A)$ and phase shift *φ* estimates can be computed as (Heiskala *et al* 2007, Heiskala 2009)

\begin{eqnarray*}\mathrm{ln}(A)=\mathrm{ln}(\sqrt{{X}^{2}+{Y}^{2}})\quad \mathrm{and}\quad \varphi =\mathrm{atan}2\left(\displaystyle \frac{Y}{X}\right),\end{eqnarray*}

respectively, with $\mathrm{atan}2$ being the four-quadrant inverse tangent function.

A complete set of measurements for a particular anatomy is simulated by considering in turns each source and recording the corresponding intensities at all sensors. Hence, a single set of measurements can be stored, e.g. in a vector ${M}_{\mathrm{MC}}\in {{\mathbb{C}}}^{{n}_{d}{n}_{s}}$, where *n*_*d*_ and *n*_*s*_ are the number of sensors (i.e. detectors) and the number of sources, respectively. That is, each source–detector pair produces a single complex number *X* + i*Y* as the corresponding measurement. Take note that the measurement vector *M*_MC_ obviously depends on the optical parameters in Ω and the measurement geometry.

#### 2.1.2. Diffusion approximation

Assuming photon input at the *k*th source, the elliptic boundary value problem corresponding to the DA in the frequency domain reads (Schweiger *et al* 1995, Arridge 1999, Heino and Somersalo 2002, Hannukainen *et al* 2015)

\begin{eqnarray*}\left\{\begin{array}{ll}-{\mathrm{\nabla }}\cdot \left(\kappa {\mathrm{\nabla }}{{\mathrm{\Phi }}}_{k}\right)+\left(\displaystyle \frac{{\mathrm{i}}\omega }{c}+{\mu }_{a}\right){{\mathrm{\Phi }}}_{k}=0\quad &amp; \mathrm{in}\,{\mathrm{\Omega }},\\ (1-\rho ){{\mathrm{\Phi }}}_{k}+2(1+\rho )\nu \cdot \kappa {\mathrm{\nabla }}{{\mathrm{\Phi }}}_{k}=4{Q}_{k}\quad &amp; \mathrm{on}\,\partial {\mathrm{\Omega }},\end{array}\right.\end{eqnarray*}

where ${{\mathrm{\Phi }}}_{k}:{\mathrm{\Omega }}\to {\mathbb{C}}$ is the photon fluence, $\omega =2\pi f$ is the angular modulation frequency, *ν* is the exterior unit normal of ∂Ω and $\kappa \in {L}_{+}^{\infty }({\mathrm{\Omega }})$ is the diffusion coefficient defined by

\begin{eqnarray*}\kappa =\displaystyle \frac{1}{3({\mu }_{a}+{\mu }_{s}^{{\prime} })},\end{eqnarray*}

with ${\mu }_{s}^{{\prime} }=(1-g){\mu }_{s}$ being the reduced scattering coefficient. We model the photon injection as a homogeneous diffuse emitter of unit strength on the boundary of the domain. This corresponds to a boundary source *Q*_*k*_ ∈ *L* ^∞^(∂Ω) that is the characteristic function of the *k*th source patch divided by its area. The parameter *ρ* ∈ [0, 1] is the effective reflection coefficient modelling the reflection of photons back toward the interior of Ω on the boundary ∂Ω in the framework of the diffusion approximation (Haskell *et al* 1994).

According to our DA model, the measurement at the *j*th sensor is defined as (cf., e.g. Heino and Somersalo 2002, Hannukainen *et al* 2015)

\begin{eqnarray*}{M}_{{jk}}={\int }_{\partial {\mathrm{\Omega }}}{P}_{j}(1-\rho )\left(\displaystyle \frac{1}{4}{{\mathrm{\Phi }}}_{k}-\displaystyle \frac{1}{2}\nu \cdot \kappa {\mathrm{\nabla }}{{\mathrm{\Phi }}}_{k}\right){\mathrm{d}}S=\displaystyle \frac{1}{2}\displaystyle \frac{1-\rho }{1+\rho }{\int }_{\partial {\mathrm{\Omega }}}{P}_{j}{{\mathrm{\Phi }}}_{k}{\mathrm{d}}S,\end{eqnarray*}

where *P*_*j*_ is the ‘device function’ of the *j*th sensor and the equality follows from the boundary condition in (4) because the sources and sensors are not allowed to overlap. In our numerical experiments, the sensor is modelled as homogeneous, which corresponds to *P*_*j*_ being the characteristic function of the circular sensor patch on ∂Ω. Overall, our choices of measurement and injection models are limited to arguably the simplest options to facilitate straightforward comparison between FE based solutions of (4)–(6) and the MC based simulations described in the previous section.

The weak formulation of (4) is to find a photon fluence Φ_*k*_ ∈ *H* ^1^(Ω) such that (cf., e.g. Hannukainen *et al* 2015)

\begin{eqnarray*}{\int }_{{\mathrm{\Omega }}}\left(\kappa {\mathrm{\nabla }}{{\mathrm{\Phi }}}_{k}\cdot {\mathrm{\nabla }}\bar{\psi }+\left(\displaystyle \frac{{\mathrm{i}}\omega }{c}+{\mu }_{a}\right){{\mathrm{\Phi }}}_{k}\,\bar{\psi }\right){\mathrm{d}}x+\displaystyle \frac{1-\rho }{\left.2(1+\rho \right)}{\int }_{{\mathrm{\partial }}{\mathrm{\Omega }}}{{\mathrm{\Phi }}}_{k}\,\bar{\psi }\,{\mathrm{d}}S=\displaystyle \frac{2}{1+\rho }{\int }_{{\mathrm{\partial }}{\mathrm{\Omega }}}{Q}_{k}\bar{\psi }\,{\mathrm{d}}S,\end{eqnarray*}

for all *ψ* ∈ *H* ^1^(Ω). To simulate the measurements, (7) is first solved for all *k* = 1, ..., *n*_*s*_ with a custom simulation tool built on top of scikit-fem (Gustafsson and McBain 2020), and then the measurements at the sensors are computed by evaluating the integrals (6) for all source-sensor pairs, i.e. for all *k* = 1, ...,* n*_*s*_ and *j* = 1, ...,* n*_*d*_. We use standard first order basis functions for the FE solutions on a tetrahedral mesh with sufficient density to represent the detailed structure of the considered head anatomies. The meshes are roughly homogeneous in density except for the area near the optoid contact areas that were refined further. As for the MC simulations, a single set of measurements is stored in a measurement vector ${M}_{\mathrm{DA}}\in {{\mathbb{C}}}^{{n}_{d}{n}_{s}}$.

### 2.2. Sensitivity profiles

Image reconstruction is dependent on the modelled 3D distribution of light within the head via the sensitivity profiles or Jacobian matrices. The Jacobians contain the derivatives of a measurable with respect to an optical parameter—or the absorption coefficient in this study—for each element (voxel or tetrahedron) in the medium. The Jacobians form the linear model used for reconstructing the distribution of haemoglobin concentration changes in the medium from the changes in the measurements (see equation (14)). Next, we introduce the computation of the Jacobians with the two forward solvers.

#### 2.2.1. Development of the frequency-domain ‘replay’ feature for MCX

The exact formulas for the derivatives of the real and imaginary components, *X* and *Y*, of the measurement for a given source and sensor with respect to the absorption coefficient *μ*_*a*,*j*_ in voxel *j* can be derived from the equations in (2) as (Heiskala *et al* 2007, Heiskala 2009)

\begin{eqnarray*}\begin{array}{rcl}\displaystyle \frac{{\mathrm{\partial }}X}{{\mathrm{\partial }}{\mu }_{a,j}} &amp; = &amp; \displaystyle \sum _{p}\displaystyle \frac{{\mathrm{\partial }}{w}_{p}}{{\mathrm{\partial }}{\mu }_{a,j}}\,\cos (2\pi {{ft}}_{p})=-\displaystyle \sum _{p}{l}_{p,j}\,{w}_{p}\,\cos (2\pi {{ft}}_{p}),\\ \displaystyle \frac{{\mathrm{\partial }}Y}{{\mathrm{\partial }}{\mu }_{a,j}} &amp; = &amp; \displaystyle \sum _{p}\displaystyle \frac{{\mathrm{\partial }}{w}_{p}}{{\mathrm{\partial }}{\mu }_{a,j}}\,\sin (2\pi {{ft}}_{p})=-\displaystyle \sum _{p}{l}_{p,j}\,{w}_{p}\,\sin (2\pi {{ft}}_{p}).\end{array}\end{eqnarray*}

The derivatives for the log-amplitude and phase shift given in (3) can be derived from these formulas as

\begin{eqnarray*}\displaystyle \frac{\partial \mathrm{ln}(A)}{\partial {\mu }_{a,j}}=\displaystyle \frac{1}{{A}^{2}}\left(X\displaystyle \frac{\partial X}{\partial {\mu }_{a,j}}+Y\displaystyle \frac{\partial Y}{\partial {\mu }_{a,j}}\right),\end{eqnarray*}

\begin{eqnarray*}\displaystyle \frac{\partial \varphi }{\partial {\mu }_{a,j}}=\displaystyle \frac{1}{{A}^{2}}\left(X\displaystyle \frac{\partial Y}{\partial {\mu }_{a,j}}-Y\displaystyle \frac{\partial X}{\partial {\mu }_{a,j}}\right).\end{eqnarray*}

We see that both derivatives can be computed from *X* and *Y* and the respective derivatives, where the essential unknowns prior to the simulation are the paths *l*_*p*,*j*_ , flight times *t*_*p*_ and weights *w*_*p*_ of the detected photons, which can all be computed using the recorded *l*_*p*,*j*_ over all voxels *j* in the medium.

**Figure 1.**
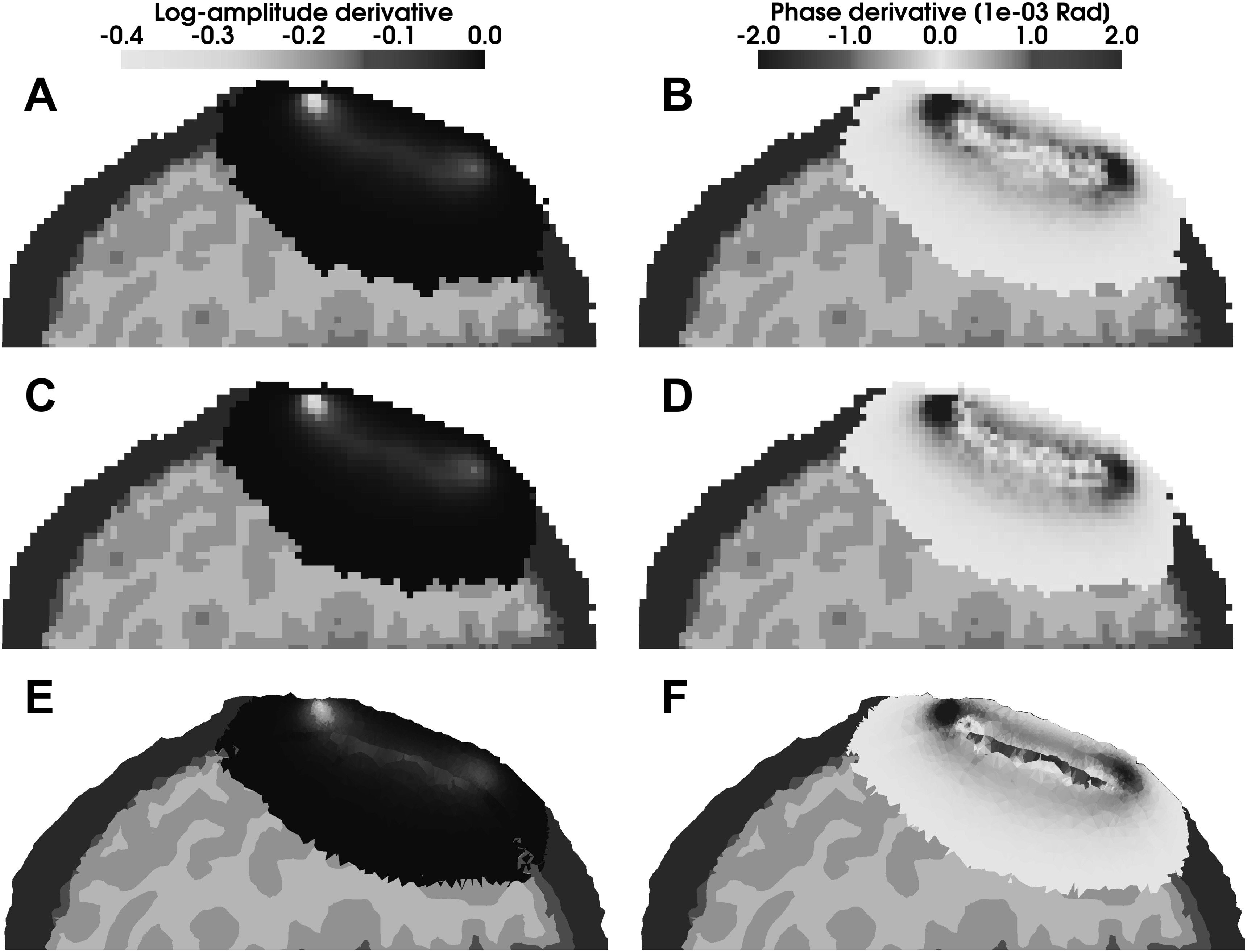
Axial slices of the log-amplitude (left column) and phase shift (right column) Jacobians for one optode pair with source–detector separation (SDS) of 28 mm in the reference head model, computed with three alternative approaches: (A), (B): MCX FD ‘rf replay’ method; (C), (D): Difference quotient estimates with MCX; (E), (F): Adjoint method implemented for DA with FEM. The derivatives are given per absorption coefficient change in 1 mm^3^ volume, thus in [mm] or [rad x mm] units, for the log-amplitude and phase shift Jacobians, respectively .

We added the ready computation of the Jacobians for both the *X* and *Y*, and the log-amplitude and phase shift to the MCX software as the new radio-frequency (rf) ‘rf replay’ feature to add to the ‘replay’ mode that originally handled CW and TD modalities (Yao *et al* 2018). An example MATLAB script for computing the *X* and *Y* Jacobians is available at: https://github.com/fangq/mcx/blob/master/mcxlab/examples/demo_mcxlab_replay.m, and for the log-amplitude and phase Jacobians at: https://github.com/fangq/mcx/blob/master/mcxlab/examples/demo_replay_frequencydomain.m. The idea of ‘replay’ is that after the forward simulation, the tissue-wise partial-path-lengths and the simulation information (random number generator seeds) are saved for the detected photons. The *t*_*p*_ and *w*_*p*_ are computed for each detected photon, and then their forward simulation is ‘replayed’ to track the *l*_*p*,*j*_ factors for computation of the Jacobians. Since the ‘rf replay’ is a new feature implemented for this study, we validated the process by comparison to the difference quotient estimates for the Jacobians. Figure 1 shows axial slices of the Jacobians for one optode pair on the top of the head with source–detector separation (SDS) of 28 mm . We can see that the ‘rf replay’ Jacobians in figures 1(A), (B) for $\mathrm{ln}(A)$ and *φ*, respectively, are in good agreement with the numerical estimates in figures 1(C), (D), respectively.

#### 2.2.2. Adjoint method for the diffusion approximation

In the framework of the DA, the Fréchet derivative with respect to the absorption coefficient can be computed using the so-called adjoint trick (Arridge and Schweiger 1995, Arridge 1999, Hannukainen *et al* 2015), which can be interpreted as a consequence of the reciprocity of light propagation. To this end, let ${{\mathrm{\Phi }}}_{j}^{* }\in {H}^{1}({\mathrm{\Omega }})$ be the unique weak solution to the adjoint problem

\begin{eqnarray*}\left\{\begin{array}{ll}-{\mathrm{\nabla }}\cdot \left(\kappa {\mathrm{\nabla }}{{\mathrm{\Phi }}}_{j}^{* }\right)+\left(\frac{{\mathrm{i}}\omega }{c}+{\mu }_{a}\right){{\mathrm{\Phi }}}_{j}^{* }=0 &amp; \mathrm{in}\,{\mathrm{\Omega }},\\ (1-\rho ){{\mathrm{\Phi }}}_{j}^{* }+2(1+\rho )\nu \cdot \kappa {\mathrm{\nabla }}{{\mathrm{\Phi }}}_{j}^{* }=4{P}_{j} &amp; \mathrm{on}\,\partial {\mathrm{\Omega }},\end{array}\right.\end{eqnarray*}

where the device function of the *j*th sensor *P*_*j*_ has now taken the role of the diffuse boundary source. The Fréchet derivative of the measurement *M*_*jk*_ in (6) with respect to the absorption coefficient in the direction *ζ* ∈ *L* ^∞^(Ω) can be evaluated as

\begin{eqnarray*}({D}_{{\mu }_{a}}{M}_{{jk}})\zeta =\displaystyle \frac{1}{4}(1-\rho ){\int }_{{\mathrm{\Omega }}}\zeta \left(\displaystyle \frac{1}{3{\left({\mu }_{a}+{\mu }_{s}^{^{\prime} }\right)}^{2}}{\mathrm{\nabla }}{{\mathrm{\Phi }}}_{k}\cdot {\mathrm{\nabla }}{{\mathrm{\Phi }}}_{j}^{\ast }-{{\mathrm{\Phi }}}_{k}{{\mathrm{\Phi }}}_{j}^{\ast }\right){\mathrm{d}}x,\end{eqnarray*}

where the main differences compared to the corresponding formula in (Hannukainen *et al* 2015), equation (2.7) are explained by the modelling of reflection at ∂Ω and the diffusion coefficient not being treated as an independent variable but as a function of *μ*_*a*_ via (5).

Note that solving (10) by the custom FEM solver does not considerably increase the computational burden as the sesquilinear form on the left-hand side of the variational formulation for (10) is the same as the one in (7) considered when solving the standard forward problem of the DA. Moreover, the perturbation directions *ζ* in (11) can be chosen as some of the basis functions representing the absorption on the FE mesh, which makes the evalution of the intergal on the right-hand side of (11) straightforward and the interpretation of the computed derivatives transparent. The log-amplitude and phase derivatives corresponding to *M*_*jk*_ with respect to additive perturbations in *μ*_*a*_ can be computed by decomposing the derivatives produced by (11) into real and imaginary parts and then applying the formulas (8) and (9).

The axial slices of the resulting log-amplitude and phase Jacobians for one source–detector pair are visualized in figures 1(E), (F), respectively. We can see that the DA Jacobians resemble the two MCX cases above in figures 1(A)–(D), but differ, for example, in the contrast of the positive strand in the phase Jacobian (figures 1(B), (D), (F)), which can result partly from the different CSF models (see section 3.2). Finally, we wish to point out that the MCX Jacobians could also be computed with the adjoint method, but this would presumably require Fourier-transforming TD ‘replay’ Jacobians over a dense time grid to capture their temporal behaviour, which introduces memory and SNR related challenges, since the number of detected photons within a very short time step can be small.

### 2.3. Approximation error method and analysis

Although atlas-based head models are convenient for practical experiments, the usage of an incorrect inner anatomy unavoidably leads to modelling errors. This error can, to a certain extent, be described as an additive noise term in the absolute measurements simulated by the employed forward model in the framework of the approximation error method (Kaipio and Somersalo 2005), which has been previously applied in DOT to other types of modelling errors in simpler geometries in, e.g. (Arridge *et al* 2006, Kolehmainen *et al* 2009, Mozumder *et al* 2013, 2014) and to scalp blood flow in the human head in (Heiskala *et al* 2012), and, in the context of electrical impedance tomography, to uncertainties in a simple head model in (Candiani *et al* 2021).

The fundamental idea of the approximation error approach is to model the uncertainties in the measurement setting as an abstract random process, which also renders the model for the measurements inherently stochastic. The available realizations of the setting—in our case the *N* = 165 atlas-based neonatal head anatomies—can thus be interpreted as samples of the underlying random process. By (numerically) solving the forward problem for all these samples, one can approximately propagate the effect of the uncertainties in the measurement setup to the measurement data. By computing statistics of the simulated measurements, one can then explicitly model the induced uncertainties in the data, e.g. as an additive Gaussian random variable, the effect of which can subsequently be accounted for within the Bayesian inversion paradigm.

To exemplify the use of the approximation error method in DOT, let us assume for simplicity that the anatomical modelling errors are the only source of noise in the measurements or that the actual measurement noise is negligible compared to the modelling-induced errors. The forward model of light transport (used in the estimation of baseline optical parameters) can be simplistically presented as

\begin{eqnarray*}\vec{y}=A(\vec{x})+\vec{e},\end{eqnarray*}

where $\vec{y}\in {{\mathbb{R}}}^{2{n}_{d}{n}_{s}}$ is a column vector carrying the log-amplitude and phase shift measurements for all source–detector pairs, $\vec{x}$ represents the unknown optical parameters of interest one would like to estimate, *A* is the nonlinear forward operator connecting the optical head model to the measurements, and $\vec{e}$ presents the approximation error noise accounting for the uncertainties in parameters of secondary interest in the forward model. Making the strong assumption that $\vec{x}$ and $\vec{e}$ are *a priori* independent, one can employ Bayes’ formula to write the posterior probability density for $\vec{x}$ as

\begin{eqnarray*}\pi (\vec{x}\,| \,\vec{y})\propto {\pi }_{\mathrm{prior}}(\vec{x})\pi (\vec{y}\,| \,\vec{x})={\pi }_{\mathrm{prior}}(\vec{x}){\pi }_{\mathrm{noise}}(\vec{y}-A(\vec{x})),\end{eqnarray*}

where *π*_prior_ and *π*_noise_ are the (prior) probability densities of the unknowns $\vec{x}$ and the approximation error noise $\vec{e}$, respectively. In consequence, if one can present the prior information on the unknowns in the form of a probability density and reliably model the distribution of the approximation error noise, then the posterior (13) can be used to deduce information on $\vec{x}$ based on the measurements $\vec{y}$. One can also take a more straightforward approach and simply use the approximated distribution of $\vec{e}$ to investigate what kind of changes in $\vec{x}$ can be detected in the measurements over the approximation error noise according to the model (12); we implement this approach for studying the effect of atlas modelling related errors on absolute measurements in the following sections.

The effect of atlas modelling errors on the linearized inverse problem of reconstructing optical parameter changes from the changes in the measured signal can also be investigated in a related manner. The linearized forward model, connecting the unknown column vector of element-wise optical parameter changes in the medium ${\mathrm{\Delta }}\overrightarrow{x}\in {{\mathbb{R}}}^{{n}_{e}}$ to the measured changes in the detected light ${\mathrm{\Delta }}\vec{y}$ is (cf. Heiskala *et al* 2012 )

\begin{eqnarray*}{\mathrm{\Delta }}\vec{y}\approx {{\bf{J}}}_{\mathrm{ref}}{\mathrm{\Delta }}\vec{x},\end{eqnarray*}

where the effect of measurement noise has been ignored and ${{\bf{J}}}_{\mathrm{ref}}\in {{\mathbb{R}}}^{2{n}_{d}{n}_{s}\times {n}_{e}}$ is the Jacobian of the forward operator with respect to $\vec{x}$ computed employing the correct head anatomy of the considered reference neonate discretized into *n*_*e*_ elements (voxels or tetrahedrons). If one approximates **J**_ref_ by a Jacobian **J** computed for an atlas anatomy, the linearized model transforms into

\begin{eqnarray*}{\mathrm{\Delta }}\vec{y}\approx {\bf{J}}{\mathrm{\Delta }}\vec{x}+({{\bf{J}}}_{\mathrm{ref}}-{\bf{J}}){\mathrm{\Delta }}\vec{x}={\bf{J}}{\mathrm{\Delta }}\vec{x}+{\vec{e}}_{{\mathrm{\Delta }}\vec{x}},\end{eqnarray*}

where we have for simplicity assumed that the discretization for the optical parameters represented by $\vec{x}$ are compatible in the reference and atlas anatomies. The term ${\vec{e}}_{{\mathrm{\Delta }}\vec{x}}=({{\bf{J}}}_{\mathrm{ref}}-{\bf{J}}){\mathrm{\Delta }}\vec{x}$ represents the error in the difference measurement—under the linearized model—caused by performing the linearization in the wrong, atlas-based anatomy. For a fixed ${\mathrm{\Delta }}\vec{x}$, the error ${\vec{e}}_{{\mathrm{\Delta }}\vec{x}}$ has an interpretation as a realization of a random variable: the Jacobian **J** is defined by a particular neonatal head, i.e. by a sample of the underlying abstract random process, giving a stochastic interpretation for ${\vec{e}}_{{\mathrm{\Delta }}\vec{x}}$ as well. If ${\mathrm{\Delta }}\vec{x}$ were also interpreted as a random variable in Bayesian inversion, it would obviously be unreasonable to assume that ${\mathrm{\Delta }}\vec{x}$ and ${\vec{e}}_{{\mathrm{\Delta }}\vec{x}}$ are independent. Hence, we settle in this work with comparing the estimated second order statistics for ${\vec{e}}_{{\mathrm{\Delta }}\vec{x}}$ to the magnitude of the reference difference signal to analyze the feasibility of reconstructing ${\mathrm{\Delta }}\vec{x}$ from difference data. If the approximation error method were to be used in Bayesian inversion in connection to (14), the correct approach would arguably be to model the Jacobian **J** itself as a random matrix and to build methods for accounting for the related uncertainty when solving (14) within the Bayesian paradigm.

Let us then more precisely explain how the second order statistics, i.e. the expected value and the covariance matrix, can be estimated for $\vec{e}$. To this end, let ${\vec{y}}_{\mathrm{ref}}$ denote the simulated reference measurements, and ${\vec{y}}_{1},...,{\vec{y}}_{N}$ denote the measurements for the atlas anatomies. The sample mean and covariance for the approximation error noise are computed as

\begin{eqnarray*}{\overrightarrow{\mu }}_{\overrightarrow{e}}=\displaystyle \frac{1}{N}\sum _{i=1}^{N}\left({\overrightarrow{y}}_{{\mathrm{ref}}}-{\overrightarrow{y}}_{i}\right),\end{eqnarray*}

\begin{eqnarray*}{{\mathrm{\Gamma }}}_{\overrightarrow{e}}=\displaystyle \frac{1}{N-1}\sum _{i=1}^{N}\left({\overrightarrow{y}}_{{\mathrm{ref}}}-{\overrightarrow{y}}_{i}-{\overrightarrow{\mu }}_{\overrightarrow{e}}\right){\left({\overrightarrow{y}}_{{\mathrm{ref}}}-{\overrightarrow{y}}_{i}-{\overrightarrow{\mu }}_{\overrightarrow{e}}\right)}^{{\mathrm{\top }}}.\end{eqnarray*}

Note that the knowledge of accurate enough estimates for the second order statistics enables forming a Gaussian approximation for the examined random variable, which is the standard technique for introducing a probability density for the approximation error in (13). The second order statistics for ${\vec{e}}_{{\mathrm{\Delta }}\vec{x}}$ corresponding to a *given* ${\mathrm{\Delta }}\vec{x}$ can be computed analogously by replacing ${\overrightarrow{y}}_{{\mathrm{ref}}}-{\overrightarrow{y}}_{i}$ by $({{\bf{J}}}_{\mathrm{ref}}-{{\bf{J}}}_{i}){\mathrm{\Delta }}\vec{x}$ in (15)–(16), with **J**_*i*_ denoting the Jacobian computed in the *i*th atlas anatomy.

It can be argued that the approximation error method, as characterized by (13), is justifiable if the covariance matrix ${{\mathrm{\Gamma }}}_{\vec{e}}$ has only a limited number of eigenvalues whose square roots are larger or of the same magnitude as the projections of the signal of interest in the measurements onto the associated eigenvectors of ${{\mathrm{\Gamma }}}_{\vec{e}}$. Such a property would imply that some source-detector pairs, or a set of their linear combinations, would cause/carry the majority of the modelling error, with other linear combinations potentially producing more informative data. Thus, in addition to observing the magnitude of the error in the absolute and difference measurements caused by the varying inner anatomy, we will also study the eigenvalues of ${{\mathrm{\Gamma }}}_{\vec{e}}$ and ${{\mathrm{\Gamma }}}_{{\vec{e}}_{{\mathrm{\Delta }}\vec{x}}}$ and compare them to the projections of the signals of interest onto the corresponding eigenvectors.

For the absolute measurements, as the signal of interest we will consider the difference in the reference measurements due to changes in the S&S or GM baseline absorption coefficient, since the segmentation and the optical parameters both affect the modelled light transport (Heiskala *et al* 2009a). For the difference measurements, we consider the change in the reference measurements due to a local absorption perturbation simulating brain activity.

## 3. Models and implementation

### 3.1. Initial preparations

For this study, we used the database of neonatal head models introduced in Collins-Jones *et al* (2021) and available at: https://www.ucl.ac.uk/dot-hub (Makropoulos *et al* 2018, Collins-Jones *et al* 2021). The database was created in collaboration between the University College London (UCL) and the Centre for the Developing Brain at King’s College London, using data from participants of the Developing Human Connectome Project. The repository contains 215 early preterm to post-term neonate models over the age range of 29.3–44.3 weeks of combined gestational and chronological age (PMA). For this study, we limited to the 193 late preterm to post-term neonates with PMA of at least 34.0 weeks. We excluded the earlier preterm subjects to limit the variation in the maturity of the brain. By 34 weeks of gestation, all major sulci have developed in most newborns; the secondary occipital sulci and the insular sulci are the last to appear around 34 weeks of gestation (Garel *et al* 2001). As explained later, 166 of these neonates were eventually included in the numerical analysis of this study.

From the database, we took the segmented 3D voxel models (tissue masks), the anatomical landmark locations (inion = Iz, nasion = Nz, left preauricular point = LPA, right preauricular point = RPA, and vertex = Cz), and the 249 cranial points according to the 10–5 system (Collins-Jones *et al* 2021). The models consist of cubic 0.5 × 0.5 × 0.5 mm^3^ voxels, and each voxel is labeled with an index from the range 0–9, corresponding to one of the nine originally segmented tissue types: combined scalp and skull (S&S), CSF, cortical grey matter, white matter, ventricles, cerebellum, deep grey matter, the brainstem and the hippocampus.

As the first step in our model creation pipeline, we performed a combined reflection and rigid transformation to undo the left–right hemisphere mirroring in the masks, transform them to the ‘head frame’ (*y*-axis runs from Iz to Nz, *x*-axis approximately from LPA to RPA, and *z*-axis runs vertically from bottom to top; Collins-Jones *et al* 2021), and fix possible head tilt due to misplaced LPA and/or RPA. For each fixed mask, we created a dense triangular surface mesh with the Iso2Mesh software (https://iso2mesh.sf.net, https://github.com/fangq/iso2mesh) (Fang and Boas 2009a, Tran *et al* 2020) by setting the maximum Delaunay sphere radius to 0.5 mm. We projected the provided cranial points to the surface mesh, and these form the basis for surface-based linear registration between two models.

We reduced the number of tissue types to five by combining cortical and deep grey matter and the hippocampus to grey matter (GM), and marking the brainstem and cerebellum as white matter (WM) (Collins-Jones *et al* 2021). The CSF in the sulci was separated from the CSF in the subarachnoid (CSF-1) and combined with the ventricles (CSF-2), as described in detail below before explaining further steps in the pipeline. The tissue-wise optical parameters were selected from literature for neonates at the near-infrared wavelength of 800 nm (Fukui *et al* 2003, Jönsson *et al* 2018), and are given in table 1.

**Table 1.** Selected literature-based optical parameters at 800 nm (Fukui *et al* 2003, Jönsson *et al* 2018) for the two considered forward solvers (MCX and DA). CSF-1 refers to the semidiffusive subarachnoid cerebrospinal fluid (CSF), and CSF-2 is the clearer CSF in the sulci and ventricles.

Tissue type	*μ*_*a*_ [mm^−1^]	*μ*_*s*_ [mm^−1^]	*g*	*n*
Scalp & skull	0.015	16	0.9	1.4
CSF-1	0.004	1.6	0.9	1.4
CSF-2	0.002 (MCX)/0.004 (DA)	0.4 (MCX)/1.6 (DA)	0.9	1.4
Grey matter	0.048	5	0.9	1.4
White matter	0.037	10	0.9	1.4

### 3.2. A new algorithm for cerebrospinal fluid segmentation

Realistic modelling of light propagation in the CSF is an important part in the overall model of the optical structure of the head (Okada *et al* 1997, Okada and Delpy 2003). Since the CSF has relatively low absorption and scattering, it offers paths along which light can travel for relatively long distances without significant loss of intensity. In addition to the thickness of S&S, the amount of CSF and the locations of the CSF-filled sulci in the brain are probably the most relevant sources of anatomy-related error when using atlas models. The assumptions underlying the DA do not hold in clear CSF, which has led to several different approaches for handling these regions: radiosity-diffusion hybrid models (Dehghani *et al* 2000, Hyvönen 2002), and modelling the CSF as a semi-diffusive substance of intermediate scattering (e.g. in Okada and Delpy 2003, Custo *et al* 2006). Nevertheless, the exact optical properties and the optimal methodology for accurate modelling of the CSF are unknown.

The pia mater surrounds the brain tissue, and the space between pia mater and arachnoid mater is called the subarachnoid tissue. Above the subarachnoid tissue resides the dura mater, skull and scalp. The subarachnoid tissue contains arachnoid trabeculae and CSF (Mortazavi *et al* 2018). The trabeculae form a mesh of fibrous tissue which gives mechanical support to the brain and breaks the subarachnoid CSF into smaller compartments, limiting the free path of the photons within the subarachnoid layer. Optical coherence tomography (OCT) has been used to investigate the cross-sectional structure of this layer in adult humans (Hartmann *et al* 2019, Benko *et al* 2020): The observed mean depth of the subarachnoid tissue was approximately 0.57 mm (Hartmann *et al* 2019). Some trabeculae extend to the space within the sulci. Yet, the OCT images do not extend deep into the space between lobes in the Sylvian fissure, for example, and thus the quantity of trabecular tissue in those parts is unknown.

Okada and Delpy (2003) investigated the optical properties of the subarachnoid CSF by comparing simulated data generated using a multilayer slab model with measured mean time data from an adult subject as a function of the source–detector separation (SDS), and estimated the density of the trabeculae and the effective scattering coefficient of the subarachnoid CSF layer (Okada and Delpy 2003). The CSF layer was assumed to be 2-mm thick. They arrived at an estimate of 0.16–0.32 mm^−1^ for the effective reduced scattering coefficient ${\mu }_{s}^{{\mathrm{{\prime} }}}$ (see equation (5)) of the subarachnoid CSF using this model. If the actual subarachnoid CSF layer is thinner (0.57 mm in Hartmann *et al* 2019) than in the model by Okada and Delpy (2003), a lower scattering coefficient would be needed to achieve the deviation from linearity observed in the mean time data.

**Figure 2.**
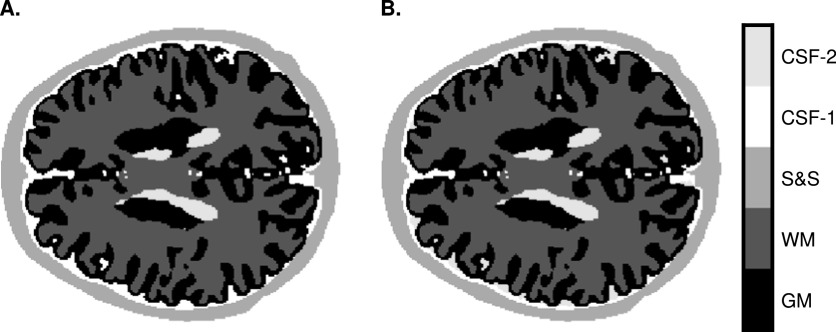
Visualization of an axial slice of a neonate head (not the reference) before (A) and after (B) separating the semidiffusive subarachnoid cerebrospinal fluid (CSF) (CSF-1; white layer) from the clearer CSF (CSF-2; yellow layer) in the brain sulci and the ventricles. The tissue types (GM = grey matter, WM = white matter, S&S = scalp and skull) have been arranged in ascending order (from top to bottom) according to the absorption coefficient.

T2-weighted magnetic resonance (MR) images show the CSF as white and T1-weighted images show it as a dark region. In many areas, the tangential layer of subarachnoid CSF above the gyri is difficult to see as a region which contrasts with its surrounding tissue, whereas the sulci in many cases show up as bright areas in the T2, and in case of larger pockets of CSF in some sulci, similar in value to the ventricles. Possible reasons for the higher T2 values in the sulci include the impact of brain pulsation on the MR imaging, and lower fractional volume of trabecular tissue and higher fractional volume of CSF. In the original UCL database models, the ventricles have been separated from the remaining CSF which surrounds the cerebral cortex. Because of above considerations, we decided to change the segmentation of the outer CSF in the UCL database and divide it to subarachnoid CSF (CSF-1) with (not reduced) *μ*_s_ = 1.6 mm^−1^ and sulcus CSF (CSF-2) with *μ*_s_ = 0.4 mm^−1^ to permit the use of a lower scattering coefficient in the CSF of the sulci compared to the outer layer of the subarachnoid tissue. The absorption coefficient was lowered by half to the same value that Okada and Delpy (2003) selected for CSF without the arachnoid trabeculae (Okada and Delpy 2003). A similar approach of low-scattering sulci and semidiffusive subarachnoid CSF was used in a previous publication (Maria *et al* 2022), where it enabled more realistic optical parameter fitting in an atlas-based model of the two-year-old child head and also helped in localizing functional activation in the insula and somatosensory cortex, consistent with adult fMRI literature. Our novel algorithm for approximately separating the subarachnoid CSF from the CSF in the sulci (and the ventricles) is explained in the following, and a visualization of the outcome is provided in figure 2.

First a dense tetrahedral mesh model is created from the segmented voxel model, with voxel side length of 0.5 mm, using the vol2mesh function in the Iso2Mesh software (Fang and Boas 2009a, Tran *et al* 2020). An imaginary interface between the semidiffusive and clearer CSF is then formed by searching for an appropriate level set in a solution to the Laplace’s equation with certain mixed Dirichlet/Neumann conditions on the boundary of the domain originally segmented as CSF. The underlying idea is to set the value of the considered solution to the Laplace’s equation to 1 everywhere on the interface between the CSF and the skull, as well as at the longitudinal fissure of the brain, and to 0 on the interface between the CSF and GM. The solution then consists of level sets that enclose the cerebral hemispheres loosely for values close to one and tightly for values close to zero. The precise choice of the value defining the diffuse and clearer CSF separating level set is, in the end, based on an expert opinion.

Due to junctions of three tissue types, e.g. along the longitudinal fissure, the aforementioned Dirichlet boundary conditions are complemented by carefully chosen Neumann boundary conditions. This is necessary to ensure the solvability of the Laplace equation in *H* ^1^, i.e. in the natural variational space for FEMs. In particular, sections with Dirichlet conditions with values 1 and 0 are not allowed to directly neighbor each other.

To allow us to be more precise, let Ω_CSF_, Ω_S&S_ and Ω_brain_ be the open subsets of the head Ω originally segmented as CSF outside the ventricles, extracerebral tissue, and grey or white matter, respectively. Furthermore, let Ω_LF_ be a thin semi-infinite plate set along the longitudinal fissure of the brain, starting in the vertical direction from the horizontal level defined by the bottom of the cerebrum and extending to infinity in horizontal and positive vertical directions. This means that $D={{\mathrm{\Omega }}}_{\mathrm{CSF}}\setminus {\overline{{\mathrm{\Omega }}}}_{\mathrm{LF}}$ essentially consist of left and right parts of Ω_CSF_ almost enclosing the respective cerebral hemispheres. We search for the solution *u* ∈ *H* ^1^(*D*) to the problem

\begin{eqnarray*}\left\{\begin{array}{rcl}{\mathrm{\Delta }}u &amp; = &amp; 0\qquad \mathrm{in}\ D,\\ u &amp; = &amp; f\qquad \mathrm{on}\ \partial D\setminus {{\mathrm{\Omega }}}_{{\mathrm{N}}},\\ \displaystyle \frac{\partial u}{\partial \nu } &amp; = &amp; 0\qquad \mathrm{on}\ \partial D\cap {{\mathrm{\Omega }}}_{{\mathrm{N}}},\end{array}\right.\end{eqnarray*}

where the Dirichlet boundary condition *f* is defined as explained above:

\begin{eqnarray*}f=\left\{\begin{array}{ll}0 &amp; \mathrm{on}\ \partial D\cap \,\partial {{\mathrm{\Omega }}}_{\mathrm{brain}},\\ 1 &amp; \mathrm{on}\ \partial D\setminus \partial {{\mathrm{\Omega }}}_{\mathrm{brain}}.\end{array}\right.\end{eqnarray*}

The vanishing Neumann boundary condition is needed in the vicinity of (erroneous) junctions of brain and skull, with Ω_LF_ interpreted as part of the skull in this context as it is associated with unit potential. More precisely, we define Ω_N_ via

\begin{eqnarray*}{{\mathrm{\Omega }}}_{{\mathrm{N}}}={N}_{a}\left(\left(\partial {{\mathrm{\Omega }}}_{{\mathrm{S}}\&amp;{\mathrm{S}}}\cap \partial {{\mathrm{\Omega }}}_{\mathrm{brain}}\right)\cup \left(\partial {{\mathrm{\Omega }}}_{\mathrm{LF}}\cap \partial {{\mathrm{\Omega }}}_{\mathrm{brain}}\right)\right),\end{eqnarray*}

where *N*_*a*_ denotes an open $a\in {{\mathbb{R}}}_{+}$ neighborhood of the considered set, that is, the set of all points that lie at a distance less than *a* from the considered set. In practice, the Neumann boundary condition can be established by simply leaving the Dirichlet boundary condition undefined for the FEM nodes corresponding to points where the skull and brain tissue or the plate along the longitudinal fissure and brain tissue meet. Such points naturally exist along ∂Ω_LF_ as the cerebral hemispheres are connected and the longitudinal fissure is not exactly planar, but they can also occur between the brain and skull due to slight discretization or segmentation errors.

Finally, the sought-for interface between the semidiffusive and clearer CSF can be defined as a level set of the solution *u* to (17), corresponding to an expert-defined value *θ* ∈ (0, 1). This results in a smooth interface mostly enclosing the cerebral hemispheres. A small threshold value leads to a thick subarachnoid CSF layer, thus bringing it deeper inside the sulci, and a large threshold value results in a thin subarachnoid CSF layer. In this work, the threshold value *θ* = 0.6 is employed, and the formed interfaces in the tetrahedral meshes of the neonatal head anatomies are used to differentiate between voxels with different types of CSF in the corresponding voxel models used with MCX.

### 3.3. Selecting the reference neonate and the optode locations

The reference neonate was selected as the term neonate with head size corresponding to the median value among the term neonates (gestational age of 37–42 weeks). We also checked that the head surface was relatively smooth and the quality of the segmentation was normal, and the tissue-wise volume ratios were close to the average over all individuals.

We used the geometry of our in-house high-density measurement probe (Jönsson *et al* 2018, Maria *et al* 2022), and placed it on the left hemisphere of the reference model; yet, the outcome of this study should not depend on the choice of the hemisphere. To begin with, we brought the flat probe close to the left hemisphere, and pressed it against the head by projecting the optodes radially towards the center of a sphere fitted to the covered surface region. The locations were further optimized with an iterative algorithm to obtain optode separations closer to the true probe geometry (Hirvi 2019, Maria *et al* 2022). Finally, we fit a sphere surface to the optode locations, and selected the center of the sphere as the target point for input light from all sources. Thus, light is initially directed to the center of curvature of the probe. The nose and mouth regions were not included in the original segmentations to ensure anonymity of the subjects, which was accounted for when placing the probe.

### 3.4. Registering the segmented voxel models to the reference exterior shape

**Figure 3.**
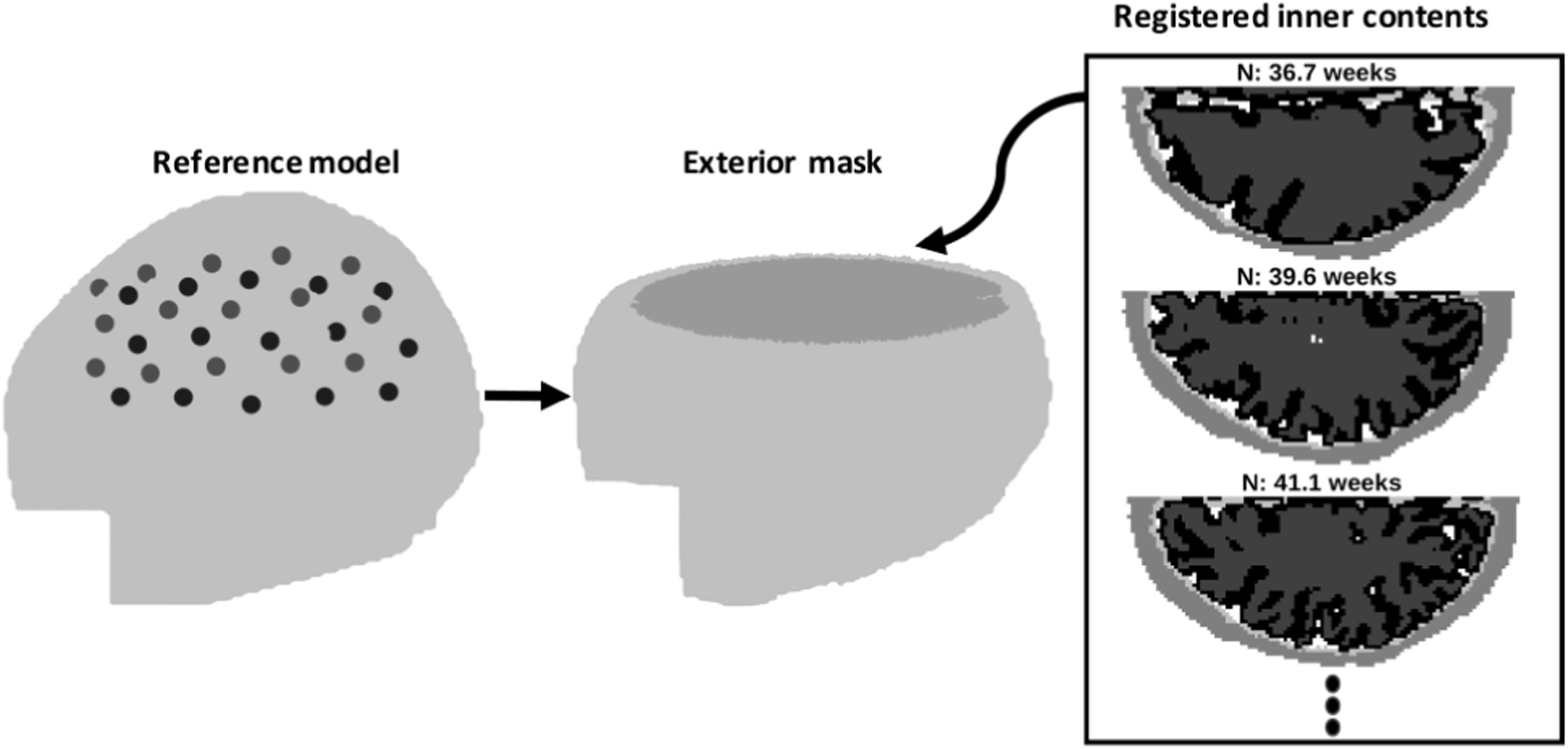
The model set creation: First select one of the neonates as the reference head shape, and place the measurement probe on its left hemisphere (source = red, detector = blue). Linearly register the other neonate models to the reference head shape, and fill the contents of the combined scalp and skull exterior mask from the reference with the registered inner contents for each neonate.

The core idea of our exact surface registration process is that we take an extracerebral S&S mask from the reference model, and fill it with the inner contents from another atlas subject’s model after it has been linearly registered to the reference head shape. Consequently, all registered models have equal head boundaries and therefore allow precisely the same positioning of the measurement probe. The procedure is visualized in figure 3. We set the thickness of the S&S mask to 2 mm, since this is the minimum S&S thickness in the reference model in the relevant region under the measurement probe. The minimum median S&S thickness in the original models under the 10–5 positions is approximately 3 mm (Collins-Jones *et al* 2021). Heiskala *et al* (2009b) reported a 5.4 mm average S&S thickness under the optodes in their neonate atlas (Heiskala *et al* 2009b). Consequently, the selected minimum thickness of 2 mm is considered justified.

The linear registration is performed in the 0.5 mm-resolution voxel models in two steps: We first solve an affine transformation (anisotropic rotation, scaling and translation) that minimizes the distance between the subject and the reference cranial points while maintaining the head in the correct orientation. Next, we solve additional anisotropic scalors for each dimension to minimize the distance between the subject and the reference surface meshes in the probe region. We perform the transformations with respect to the Nz (nasion), and initially match the subject Nz to the reference Nz. This approach was selected, since Dr Collins-Jones confirmed that the Nz was probably the ‘least ambiguous’ to determine from MRI data.

To be more exact, the affine transformation is solved to minimize the registration error ($\mathrm{RE}$) computed as (modified from Koikkalainen and Lötjönen 2004, Hirvi 2019)

\begin{eqnarray*}\mathrm{RE}=\mathrm{SRE}+{\gamma }_{1}\cdot {\mathrm{LRE}}_{1}+{\gamma }_{2}\cdot {\mathrm{LRE}}_{2},\end{eqnarray*}

where $\mathrm{SRE}$ (surface registration error) is the mean squared distance between all subject and reference cranial surface points (249 from 10–5 system + 5 landmarks), and ${\mathrm{LRE}}_{1}$ and ${\mathrm{LRE}}_{2}$ (landmark registration error) control the mean squared distance between landmarks, and thus the head orientation. ${\mathrm{LRE}}_{1}$ and ${\mathrm{LRE}}_{2}$ correspond to Iz and Nz, and LPA and RPA landmarks, respectively, with weights *γ*_1_ = 0.4–0.5 and *γ*_2_ = 0.5–0.6 depending on the visually evaluated relative goodness of the two landmarks pairs. The optimal transformation is solved with MATLAB’s backslash-operator from

\begin{eqnarray*}{\bf{W}}\left[\begin{array}{cccccccccccc}{x}_{1} &amp; {y}_{1} &amp; {z}_{1} &amp; 1 &amp; 0 &amp; 0 &amp; 0 &amp; 0 &amp; 0 &amp; 0 &amp; 0 &amp; 0\\ 0 &amp; 0 &amp; 0 &amp; 0 &amp; {x}_{1} &amp; {y}_{1} &amp; {z}_{1} &amp; 1 &amp; 0 &amp; 0 &amp; 0 &amp; 0\\ 0 &amp; 0 &amp; 0 &amp; 0 &amp; 0 &amp; 0 &amp; 0 &amp; 0 &amp; {x}_{1} &amp; {y}_{1} &amp; {z}_{1} &amp; 1\\ {x}_{2} &amp; {y}_{2} &amp; {z}_{2} &amp; 1 &amp; 0 &amp; 0 &amp; 0 &amp; 0 &amp; 0 &amp; 0 &amp; 0 &amp; 0\\ \vdots &amp; \vdots &amp; \vdots &amp; \vdots &amp; \vdots &amp; \vdots &amp; \vdots &amp; \vdots &amp; \vdots &amp; \vdots &amp; \vdots &amp; \vdots \\ 0 &amp; 0 &amp; 0 &amp; 0 &amp; 0 &amp; 0 &amp; 0 &amp; 0 &amp; {x}_{258} &amp; {y}_{258} &amp; {z}_{258} &amp; 1\end{array}\right]\vec{a}={\bf{W}}\ \left[\begin{array}{c}{x}_{1}^{\mathrm{ref}}\\ {y}_{1}^{\mathrm{ref}}\\ {z}_{1}^{\mathrm{ref}}\\ {x}_{2}^{\mathrm{ref}}\\ \vdots \\ {z}_{258}^{\mathrm{ref}}\end{array}\right],\end{eqnarray*}

where *x*_i_, *y*_i_, *z*_i_ and ${x}_{{\mathrm{i}}}^{\mathrm{ref}},{y}_{{\mathrm{i}}}^{\mathrm{ref}},{z}_{{\mathrm{i}}}^{\mathrm{ref}}$ are the coordinates of the *i*th cranial point for the subject and the reference, respectively. Vector $\vec{a}\in {{\mathbb{R}}}^{12}$ has the 12 elements of the unknown ${{\mathbb{R}}}^{3\times 4}$ affine transformation matrix, and the diagonal weight matrix ${\bf{W}}\in {{\mathbb{R}}}^{774\times 774}$ performs averaging by dividing with the square root of the number of observations in each term ($\mathrm{SRE}$ or $\mathrm{LRE}$) and also carries the square roots of the *γ*-weights. After this transformation, we find the subject’s surface mesh nodes in the probe region, and their closest nodes on the reference mesh. We form a similar matrix equation as above for solving three anisotropic scaling factors to minimize the squared distance between the surface meshes in the probe region. The affine transformation reduces the mean root-mean-squared-error (RMSE) in the distances from subject to reference cranial points over all registered subjects from (mean ± standard deviation) 6.7 ± 2.9 mm to 3.2 ± 1.0 mm, and the additional scaling further reduces the mean RMSE in the probe region from 1.9 ± 0.8 mm to 1.5 ± 0.4 mm.

Filling the inner contents of the reference 2 mm S&S mask with the registered (warped) voxel model unavoidably results in that some S&S voxels are added and some tissue voxels are removed from the intact, linearly registered model: extra external bumps filled with S&S may appear, and cavities with boundary inside the intact registered model are cut out. We set the following requirements for a ‘successful-enough’ registration: (1) after the linear registration, the nonzero regions of the registered and the reference model can only differ in S&S and CSF-1 voxels in the probe region, and there must be maximum 10 mm^3^ voxels difference in CSF-1, and (2) no WM voxels can be removed from the probe region when cutting the registered model to fit in the reference mask. Thus, there is always GM between the S&S and the WM. Following this requirement, the number of registered atlas models included to further analysis reduced to 165. Within these 165 neonates, the original range of head circumferences, and Nz–Iz and RPA–LPA distances along the head surface are 27.9–38.6 cm (mean ± standard deviation = 33.4 ± 2.1 cm), 19.0–26.9 cm (22.6 ± 1.3 cm) and 20.3–25.9 cm (23.3 ± 1.4 cm), respectively, whereas the corresponding metrics for the reference model are 33.9 cm, 22.5 cm and 23.8 cm. Thus, there is at maximum a 17.6% change (range = −17.6 to +13.9%) in the head circumference of a subject due to the registration. On average 8 ± 16 mm^3^ voxels of cortical GM were lost during the nonlinear registration due to the individual grooves on the reference surface. We visualized six axial slices from the imaged region for each registered model to check the registered segmentation quality.

The reference and the 165 registered models are resized into voxel side length of 1 mm for computational efficiency and higher voxel-wise SNR. We use the Iso2Mesh software (Fang and Boas 2009a, Tran *et al* 2020) to create the corresponding tetrahedral mesh models with maximum Delaunay sphere radius of 0.85 mm and maximum tetrahedron volume of 1.5 mm^3^. With this density, the contents of the voxel- and mesh-based segmentation look similar to eye. The meshes are refined near the optodes.

We compute the forward measurement estimates and the Jacobians for the reference and each registered voxel and tetrahedral mesh model with the MC and the DA forward solvers, respectively, and proceed to analyze the magnitude of the atlas modelling related approximation error.

## 4. Results

We will first consider the atlas modelling related changes in the simulated absolute measurements, and then in the linearized difference measurements, as described in section 2.3. We consider the log-amplitude and phase measurements separately in order to highlight the qualitative differences of the quantities. Therefore both absolute and relative measurements considered in (15)–(16) correspond to real-valued vectors carrying either log-amplitude or phase information. If not mentioned otherwise, we use the two-compartment CSF segmentation according to table 1 in the MCX simulations.

### 4.1. Absolute measurements

**Figure 4.**
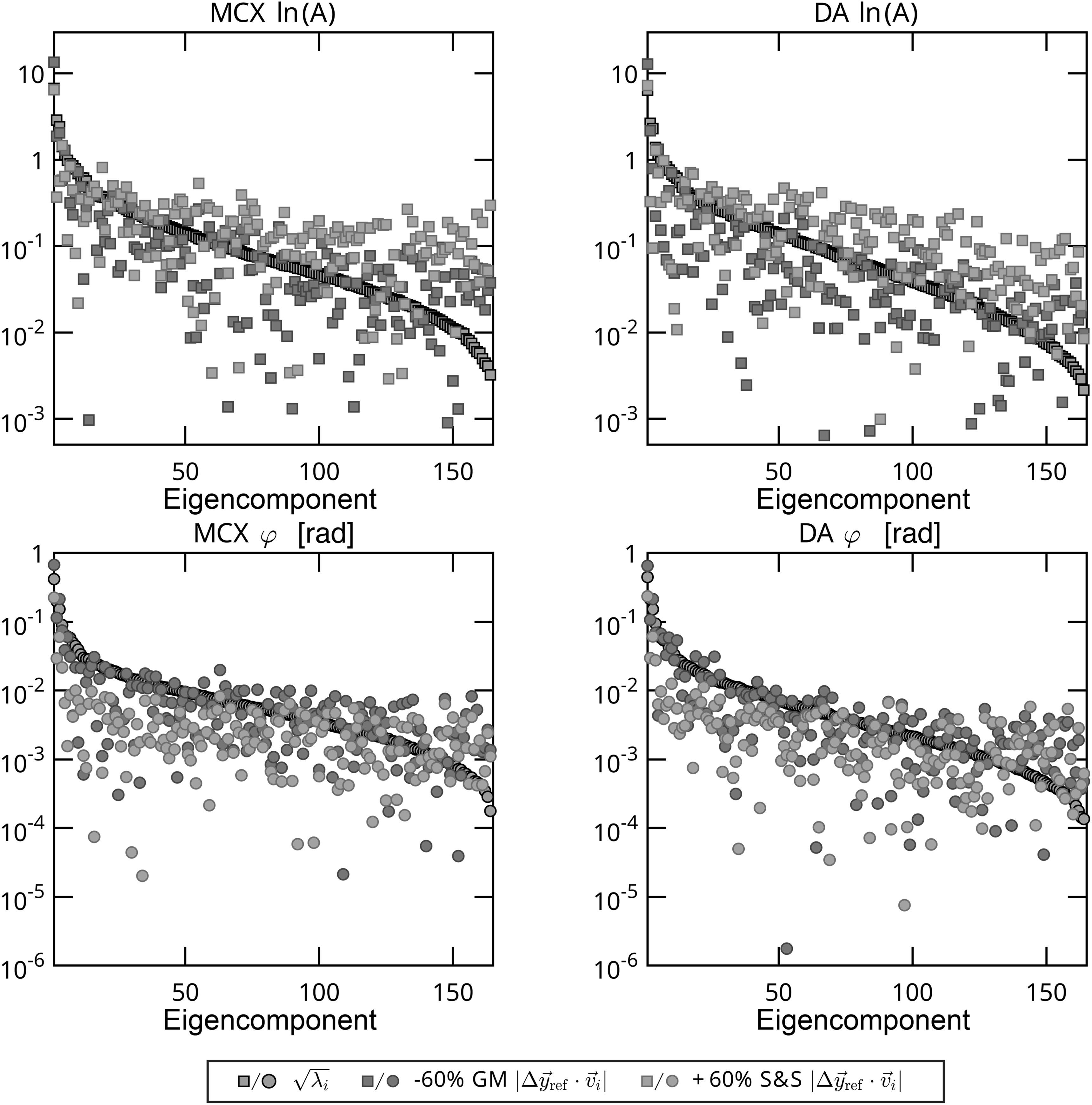
The square roots of the eigenvalues for the covariance matrix of the anatomy-induced approximation error noise compared to changes in the measurement signals caused by tissue-wise relative changes in the absorption coefficient in the reference model: −60% for the grey matter (GM) or +60% for the scalp and skull (S&S). The red and blue markers depict the absolute values of the projections of the measurement signals on the eigenvectors of the covariance matrix in the same order as the square roots of the eigenvalues are shown. All four combinations of forward solvers (MCX and DA) and measurements (log-amplitude and phase) are considered.

To begin with, denote by *λ*_1_ ≥ *λ*_2_ ≥ ⋯ ≥ *λ*_*N*−1_ > 0 the positive eigenvalues and by ${\vec{v}}_{1},...,{\vec{v}}_{N-1}\in {{\mathbb{R}}}^{m}$ the corresponding orthonormal eigenvectors of the covariance matrix ${{\mathrm{\Gamma }}}_{\vec{e}}$ computed according to (16) for either log-amplitude or phase measurements (*N*=165). In all our simulations with both MCX and the DA, the relative log-amplitude or phase measurement vectors ${\vec{y}}_{\mathrm{ref}}-{\vec{y}}_{i}$, *i* = 1, ..., *N*, were linearly independent leading to the rank of ${{\mathrm{\Gamma }}}_{\vec{e}}$ being one less than the number of the summand rank-one matrices in (16), which also explains the number of positive eigenvalues for ${{\mathrm{\Gamma }}}_{\vec{e}}$. The orthogonal projection of ${{\mathbb{R}}}^{m}$ onto the span of the eigenvectors ${\vec{v}}_{1},...,{\vec{v}}_{N-1}$ is denoted by *P*_*v*_. Take note that we are actually considering four sets of eigenvalues and associated orthonormal eigenvectors, corresponding to the four possible combinations of the solution method (MCX or the DA) and the type of measurement (log-amplitude or phase). However, for the ease of notation we do not explicitly differentiate between those in the following explanation. The square roots of the approximation error eigenvalues are illustrated on a logarithmic scale in figure 4 for the aforementioned four combinations, indicating an exponential convergence to zero with about 12 largest eigenvalues corresponding to 95% of their total sum. Note that the square root of an eigenvalue of ${{\mathrm{\Gamma }}}_{\vec{e}}$ corresponds to the standard deviation of the approximation error noise in the direction of the associated eigenvector.

The approximate covariance in (16) can be used as an estimate for the second order statistics of the approximation error noise, assuming that the number of anatomical samples is large enough and sufficiently representative of the anatomical variation. More precisely, we consider the variation in the absolute measurement vectors $\vec{y}\in {{\mathbb{R}}}^{m}$, with *m* = 186. The number of individual measurements *m* in each measurement vector arises from restricting the considered source–detector pairs to those with SDS of less than 55 mm, from the total count of 15 detectors and 15 sources, or 225 source–detector pairs. The square root of the expected squared Euclidean norm of the variation in the absolute measurement $\vec{y}$, caused by the varying anatomy, can then be estimated as

\begin{eqnarray*}{\mathrm{STD}}_{\vec{e}}=\mathrm{tr}{\left({{\mathrm{\Gamma }}}_{\vec{e}}\right)}^{1/2}={\left(\displaystyle \sum _{i=1}^{N-1}{\lambda }_{i}\right)}^{1/2}\end{eqnarray*}

because the eigenvalue *λ*_*i*_ corresponds to the variance of $\vec{e}$ in the direction of *${\vec{v}}_{i}$*. As *N* − 1 < *m*, ${\mathrm{STD}}_{\vec{e}}$ cannot account for the expected anatomy-induced error in the direction of the excluded (*m* − *N* + 1)-dimensional subspace of ${{\mathbb{R}}}^{m}$, but motivated by the exponential decay of the eigenvalues in figure 4, we work under the hypothesis that ${\mathrm{STD}}_{\vec{e}}$ anyway gives a reasonable estimate for the size of the anatomy-induced error in absolute measurements. The left-hand column of table 2 presents the ratio

\begin{eqnarray*}\displaystyle \frac{\parallel {P}_{v}{\vec{\mu }}_{\vec{e}}\parallel }{{\mathrm{STD}}_{\vec{e}}}\end{eqnarray*}

for the four combinations of forward solvers and measurement types, with the aim of comparing the expected shift in the measurements due to the mismodelled anatomy with the variation around that mean due to the same error source. In the shift we only account for the component of ${\vec{\mu }}_{\vec{e}}$ in the span of the eigenvectors of ${{\mathrm{\Gamma }}}_{\vec{e}}$ with positive eigenvalues to allow a fair comparison with ${\mathrm{STD}}_{\vec{e}}$ that only measures the anatomy-induced variations of the measurements in that subspace. However, the effect of including the projection *P*_*v*_ in (22) is minimal on the values listed in table 2 as well as in all following considerations, since it decreases the magnitude of the considered vectors by 0.005–0.37%. The values of (22) in the left-hand column of table 2 indicate that our reference head represents relatively well the anatomical average from the standpoint of log-amplitude and phase measurements of DOT: the deviation measure ${\mathrm{STD}}_{\vec{e}}$ is two to three times larger than the Euclidean norm of the expected anatomy-induced shift ${\vec{\mu }}_{\vec{e}}$.

**Table 2.** The ratios in (22) and (23) of which the latter for both a −60% perturbation in the absorption of the grey matter (GM) and a + 60% perturbation in the absorption of the combined scalp and skull layer (S&S). The values are presented for all six combinations of forward solvers (MCX and DA), cerebrospinal fluid (CSF) models (two-compartment model for MCX and all-semidiffusive CSF for MCX and DA) and measurement types (log-amplitude and phase).

	$\tfrac{\parallel {P}_{v}{\vec{\mu }}_{\vec{e}}\parallel }{{\mathrm{STD}}_{\vec{e}}}$	$\tfrac{\parallel {P}_{v}{\mathrm{\Delta }}{\vec{y}}_{\mathrm{ref}}\parallel }{{\mathrm{STD}}_{\vec{e}}}$ (GM)	$\tfrac{\parallel {P}_{v}{\mathrm{\Delta }}{\vec{y}}_{\mathrm{ref}}\parallel }{{\mathrm{STD}}_{\vec{e}}}$ (S&S)
MCX $\mathrm{ln}(A)$	0.32	1.67	0.86
MCX $\mathrm{ln}(A)$ (1 CSF type)	0.31	1.88	0.97
DA $\mathrm{ln}(A)$	0.31	1.65	0.99
MCX *φ*	0.39	1.39	0.45
MCX *φ* (1 CSF type)	0.43	1.46	0.48
DA *φ*	0.47	1.29	0.45

Let us then investigate how measurement changes induced by varying a tissue-wise absorption level compare to the errors caused by mismodelling of the head anatomy. To this end, we perturb the absorption coefficients of GM and S&S listed in table 1 in turns, and simulate the corresponding measurements for the reference anatomy. We then compare the anatomy-induced approximation error noise to the resulting signal ${\mathrm{\Delta }}{\vec{y}}_{\mathrm{ref}}={\vec{y}}_{\mathrm{pert}}-{\vec{y}}_{\mathrm{ref}}$, where ${\vec{y}}_{\mathrm{pert}}$ denotes the absolute measurement in the reference anatomy corresponding to a perturbed absorption level for one of the two considered tissue types. The quantity $| {\mathrm{\Delta }}{\vec{y}}_{\mathrm{ref}}\cdot {\vec{v}}_{i}| $ gives the magnitude of the projection of the signal ${\mathrm{\Delta }}{\vec{y}}_{\mathrm{ref}}$ onto the direction of the *i*th eigenvector of ${{\mathrm{\Gamma }}}_{\vec{e}}$. Comparing it to the square root of the corresponding eigenvalue *λ*_*i*_ indicates whether the change in the absorption level of the considered tissue or the uncertainty in the head anatomy is expected to cause a larger perturbation in the measurement in the direction of *${\vec{v}}_{i}$*. Furthermore, the ratio of the square roots of the sums of the squared projections to the (non-squared) eigenvalues, given as

**Figure 5.**
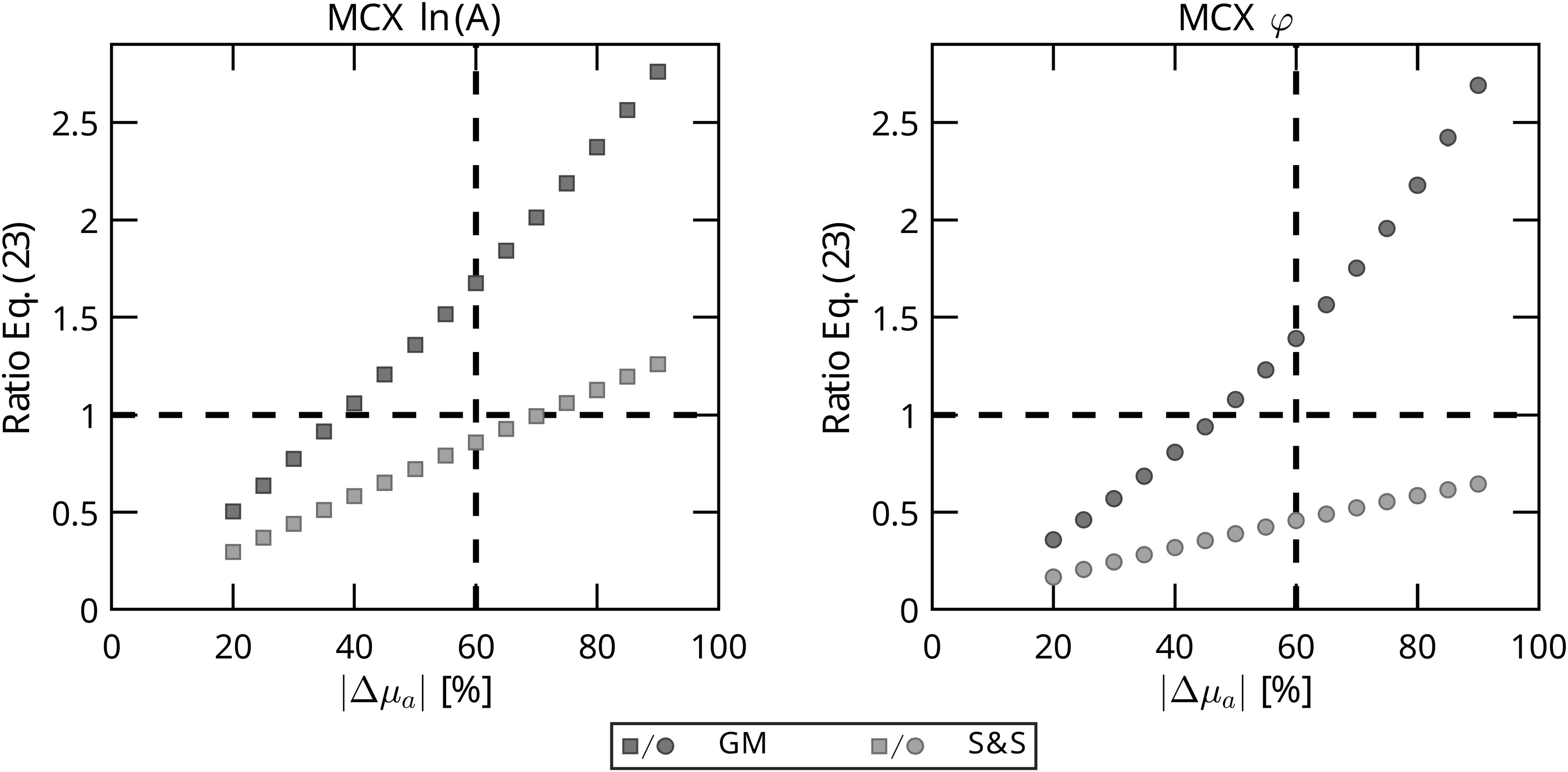
The ratios in (23) for a 20%–90% decrease and a 20%–90% increase in the absorption coefficient of grey matter (GM) and the combined scalp and skull layer (S&S), respectively. The curves are shown for both data types using the MCX forward solver.

\begin{eqnarray*}\frac{\parallel {P}_{v}{\mathrm{\Delta }}{\vec{y}}_{\mathrm{ref}}\parallel }{{\mathrm{STD}}_{\vec{e}}},\end{eqnarray*}

approximates the overall visibility of the absorption change over the anatomical error in the measurements. Figure 5 presents the ratios (23) over a 20%–90% decrease, and a 20%–90% increase in steps of 5% in the absorption coefficient of GM and S&S, respectively, for MCX and both considered data types. All four curves increase approximately linearly with respect to increasing relative absolute change in the absorption, and suggest that a ∼60% baseline change is sufficient to reach the level of the anatomical error variation for log-amplitude measurements in both tissue types. The right-hand side of table 2 gives the exact values of the ratios (23) at a 60% decrease in GM and a 60% increase in S&S absorption, and figure 4 compares the corresponding projections to the square roots of the eigenvalues over all eigenpairs. For log-amplitude measurements, the magnitude of the projection along the first eigenvector overlaps (MCX) or exceeds (DA) the corresponding eigenvalue in figure 4, and the ratios (23) in table 2 are close to one, confirming that the considered perturbations in the tissue-wise absorption levels cause changes that are comparable in size to those caused by the anatomical uncertainties. For phase measurements, the effect of the absorption change in the GM is pronounced compared to that in the S&S. The results produced by MCX and the DA are in a good qualitative agreement.

Table 2 also gives the ratios (23) for MCX with the alternative one compartment, all-semidiffusive CSF model, as for DA in table 1 (all CSF-1). The ratios on the right-hand side of table 2 are larger in the one-compartment case. Especially for the log-amplitude values, the difference is mainly due to the 11.2% increase in ${\mathrm{STD}}_{\vec{e}}$ in the two-compartment case, when the sulci (and ventricles) are modelled clearer. A possible explanation for the increase is that the contribution of the variation in the locations of the sulci to the atlas modelling related error is amplified when the sulci are more transmittive.

**Figure 6.**
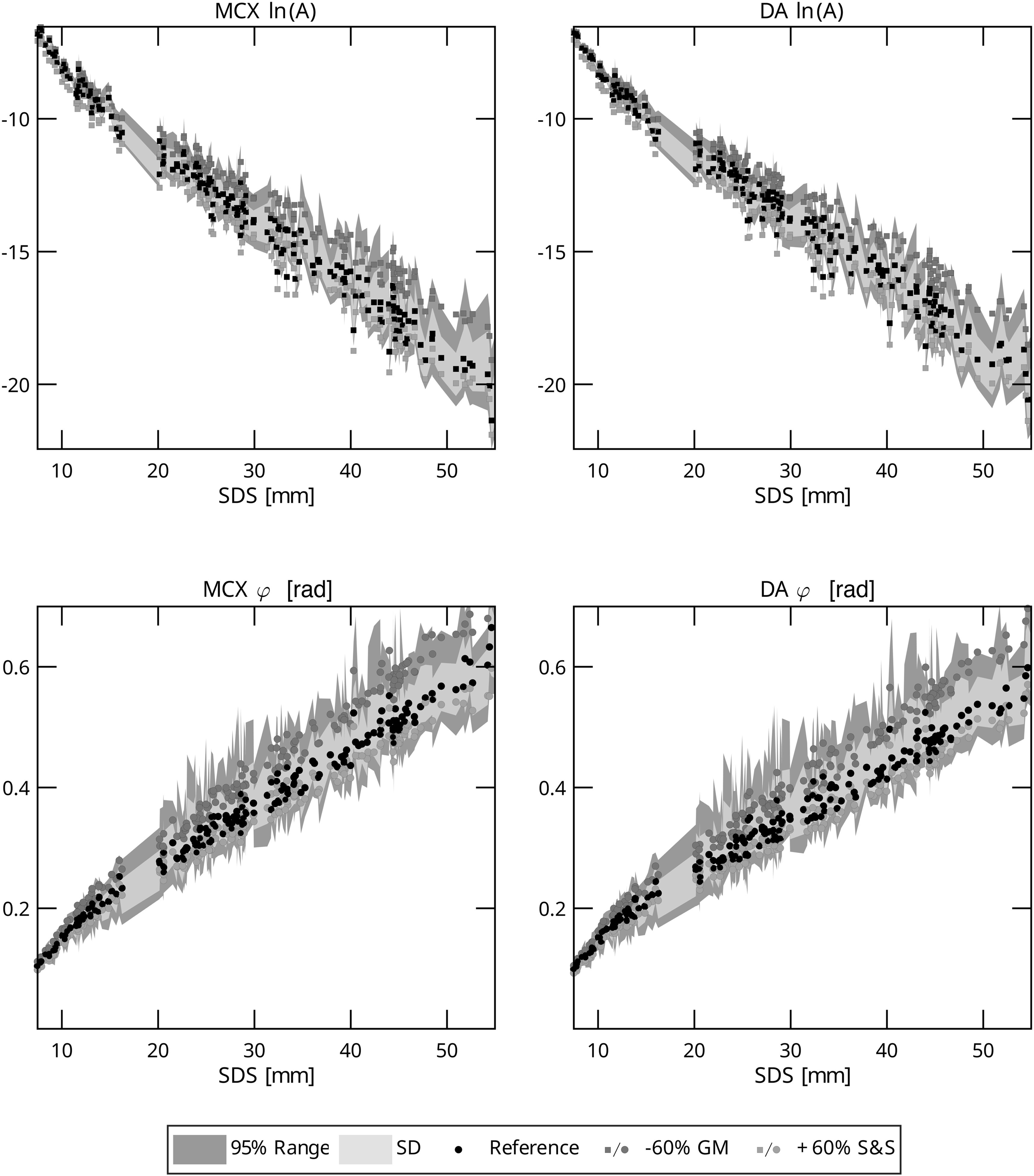
The simulated measurements versus the source-detector separation (SDS) in the reference model compared to the standard deviation (SD) and a 95% range of the measurements for the 165 registered models. The labels -60% GM and +60% S&S refer, respectively, to measurements corresponding to relative changes in the absorption coefficient of the entire grey matter (GM) and combined scalp and skull tissue (S&S) in the reference model. All four combinations of forward solvers (MCX and DA) and measurements (log-amplitude and phase) are considered.

To complete this section, figure 6 visualizes the simulated log-amplitude and phase measurements (black markers) as functions of SDS in the reference anatomy, and compares them (i) to the corresponding measurements with the selected 60% change in the absorption of GM (red markers) or S&S (blue markers), and (ii) to the variation in the measurements due to the anatomical uncertainty (cf. table 2 and figure 4). The light and dark grey envelopes represent, respectively, the standard deviation for the approximation error noise $\vec{e}$ around ${\vec{y}}_{\mathrm{ref}}-{\vec{\mu }}_{\vec{e}}$ and a range within which 95% of the measured values for the different anatomies lie (see (15) and (16)).

Figure 6 supports the findings of table 2 and figure 4: the variations in the measurements caused by the different changes in the model are comparable in size, with the absorption of S&S affecting the phase measurements less than that of GM. Furthermore, the contribution of the GM parameters seems to become more significant for both measurement types when the SDS increases, while the relative contribution of the S&S change is most significant with small SDS. The results for MCX and the DA are once again in a good agreement. The plots in figure 6 also show that many measurements within the standard deviation envelope are closer to the original reference measurements than at least the grey matter modified measurements. This implies that the corresponding neonates’ segmentations could be used as atlas models for baseline parameter fitting with some accuracy, using the same approach as in Maria *et al* (2022).

### 4.2. Difference measurements

In all experiments documented in this section, the perturbation ${\mathrm{\Delta }}\vec{x}$ in (14) corresponds to an 0.005 mm^−1^ increase in the absorption of a ball of radius 5 mm located mainly inside GM (slightly intersects the CSF or WM in some of the considered anatomies). The 10% increase was selected as a physiologically reasonable haemodynamic response to neuronal activity (Heiskala *et al* 2009b). We observed that the corresponding change in the absolute measurements for the reference neonate ${\mathrm{\Delta }}{\vec{y}}_{\mathrm{ref}}$ is not detectable over the atlas-related variation in the absolute measurements. Next we consider the second order statistics for the ‘relative linearized’ approximation error noise ${\vec{e}}_{{\mathrm{\Delta }}\vec{x}}$ according to (15)–(16) and the explanation succeeding those formulas.

Denote by *η*_1_ ≥ *η*_2_ ≥ ⋯ ≥ *η*_*N*−1_ > 0 and ${\vec{w}}_{1},...,{\vec{w}}_{N-1}$, respectively, the positive eigenvalues and the associated orthonormal eigenvectors of the ‘difference’ covariance matrix ${{\mathrm{\Gamma }}}_{{\vec{e}}_{{\mathrm{\Delta }}\vec{x}}}$ formed in accordance with (16). The orthogonal projection of ${{\mathbb{R}}}^{m}$ onto the span of the eigenvectors ${\vec{w}}_{1},...,{\vec{w}}_{N-1}$ is denoted by *P*_*w*_. The reason for the positivity of the first *N*−1 eigenvalues of ${{\mathrm{\Gamma }}}_{{\vec{e}}_{{\mathrm{\Delta }}\vec{x}}}$ is analogous to the case of absolute measurements considered in the previous section. Again, the projection from ${{\mathbb{R}}}^{m}$ to ${{\mathbb{R}}}^{N-1}$ only decreases the magnitude of the sample means ${\vec{\mu }}_{{\vec{e}}_{{\mathrm{\Delta }}\vec{x}}}$ and the projections ${P}_{w}{\mathrm{\Delta }}{\vec{y}}_{\mathrm{ref}}$ by 2 × 10^−7^—2 × 10^−5^ %. Furthermore, as for the absolute measurements, we use the same notation for the eigensystems of the four variants of ${{\mathrm{\Gamma }}}_{{\vec{e}}_{{\mathrm{\Delta }}\vec{x}}}$ corresponding to all combinations of the MCX or DA simulations and log-amplitude or phase measurements. We once again interpret

**Figure 7.**
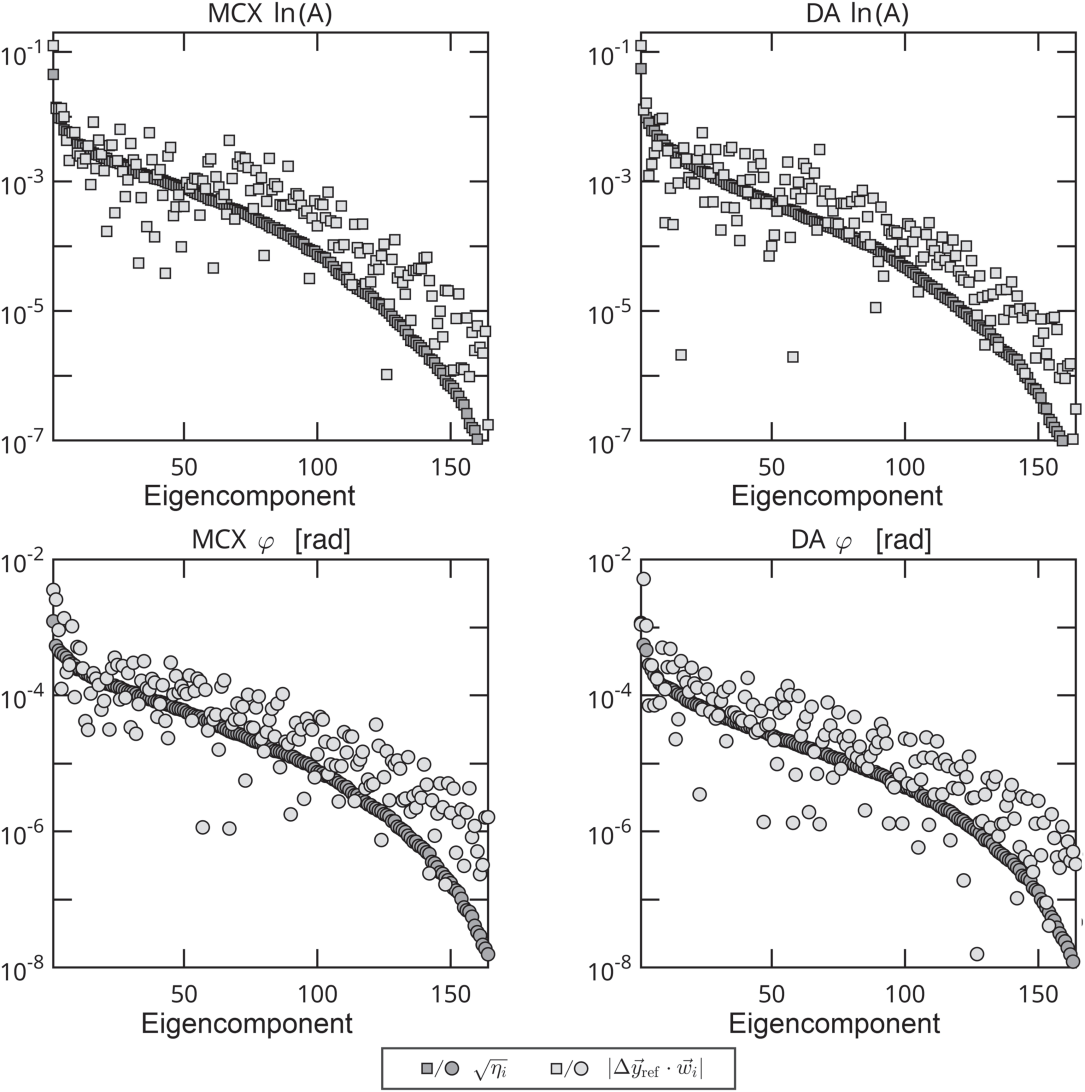
The square roots of the eigenvalues for the covariance matrix of the anatomy-induced relative approximation error noise compared to the difference measurement signal caused by a physiologically reasonable local increase in the brain absorption. The light grey squares and circles depict the absolute values of the projections of the measurement signal onto the eigenvectors of the covariance matrix in the same order as the square roots of the eigenvalues are depicted. All four combinations of forward solvers (MCX and DA) and measurements (log-amplitude and phase) are considered.

\begin{eqnarray*}{\mathrm{STD}}_{{\vec{e}}_{{\mathrm{\Delta }}\vec{x}}}=\mathrm{tr}{\left({{\mathrm{\Gamma }}}_{{\vec{e}}_{{\mathrm{\Delta }}\vec{x}}}\right)}^{1/2}={\left(\displaystyle \sum _{i=1}^{N-1}{\eta }_{i}\right)}^{1/2},\end{eqnarray*}

as a measure of the uncertainty in the difference measurements corresponding to ${\mathrm{\Delta }}\vec{x}$ caused by the anatomical variations. The square roots of the eigenvalues *η*_1_, ...,* η*_*N*−1_ are shown on a logarithmic scale for the considered four cases in figure 7; they exhibit an exponential decay in all cases, with about 16 largest eigenvalues corresponding to 95% of their total sum. The figure also compares the square roots of the approximation error eigenvalues with the corresponding absolute values of the projections of the relative reference difference measurements ${\mathrm{\Delta }}{\vec{y}}_{\mathrm{ref}}$ onto the eigenvectors of ${{\mathrm{\Gamma }}}_{{\vec{e}}_{{\mathrm{\Delta }}\vec{x}}}$, i.e. with the quantities $| {\mathrm{\Delta }}{\vec{y}}_{\mathrm{ref}}\cdot {\vec{w}}_{i}| $, *i* = 1, ...,* N*−1. As with the absolute measurements, we restricted the considered source–detector pairs to those with SDS of less than 55 mm.

Table 3 lists the ratios

\begin{eqnarray*}\displaystyle \frac{\parallel {\mathrm{\Delta }}{\vec{y}}_{\mathrm{ref}}\parallel }{\parallel {\vec{\mu }}_{{\vec{e}}_{{\mathrm{\Delta }}\vec{x}}}\parallel }\qquad \mathrm{and}\qquad \displaystyle \frac{\parallel {P}_{w}{\mathrm{\Delta }}{\vec{y}}_{\mathrm{ref}}\parallel }{{\mathrm{STD}}_{{\vec{e}}_{{\mathrm{\Delta }}\vec{x}}}}\end{eqnarray*}

for the four combinations of simulation methods and measurement types. The first of these indicators compares the size of the difference measurement in the reference setup to the magnitude of the expected shift in the difference measurement caused by the anatomy-induced variations in the Jacobian. The latter corresponds to comparing the magnitude of the reference difference measurement to the deviation of the difference measurement around its mean due to anatomical uncertainties. As all numbers in table 3 are clearly larger than one, the strength of the measurement signal caused by ${\mathrm{\Delta }}\vec{x}$ seems to be detectable over the approximation error noise caused by the geometric anatomical variations. This conclusion is also supported by figure 7 that demonstrates that the signal produced by ${\mathrm{\Delta }}\vec{x}$ is ‘visible’ over the approximation error noise in many of the eigendirections. These same conclusions can be drawn from both the MCX and the DA simulations.

**Table 3.** The ratios (24) for all four combinations of forward solvers (MCX and DA) and measurements (log-amplitude and phase).

	$\tfrac{\parallel {\mathrm{\Delta }}{\vec{y}}_{\mathrm{ref}}\parallel }{\parallel {\vec{\mu }}_{{\vec{e}}_{{\mathrm{\Delta }}\vec{x}}}\parallel }$	$\tfrac{\parallel {P}_{w}{\mathrm{\Delta }}{\vec{y}}_{\mathrm{ref}}\parallel }{{\mathrm{STD}}_{{\vec{e}}_{{\mathrm{\Delta }}\vec{x}}}}$
MCX $\mathrm{ln}(A)$	3.19	2.48
DA $\mathrm{ln}(A)$	2.54	2.14
MCX *φ*	3.65	2.67
DA *φ*	4.71	3.60

To complete this section, let us visualize how the sensitivities of the log-amplitude and phase measurements are affected by the anatomical variations in the head atlas. To this end, consider a single source-detector pair at a distance 25 mm from each other and a narrow cylinder of radius 2.5 mm extending in the normal direction 20 mm deep into the interior of the head at the midpoint between the source and the detector. We divide the cylinder into thin slices that are perpendicular to its axis and consider in turns the derivative of the (single) measurement with respect to a change in the absorption of each slice. That is, for each slice indexed by *j* we compute

**Figure 8.**
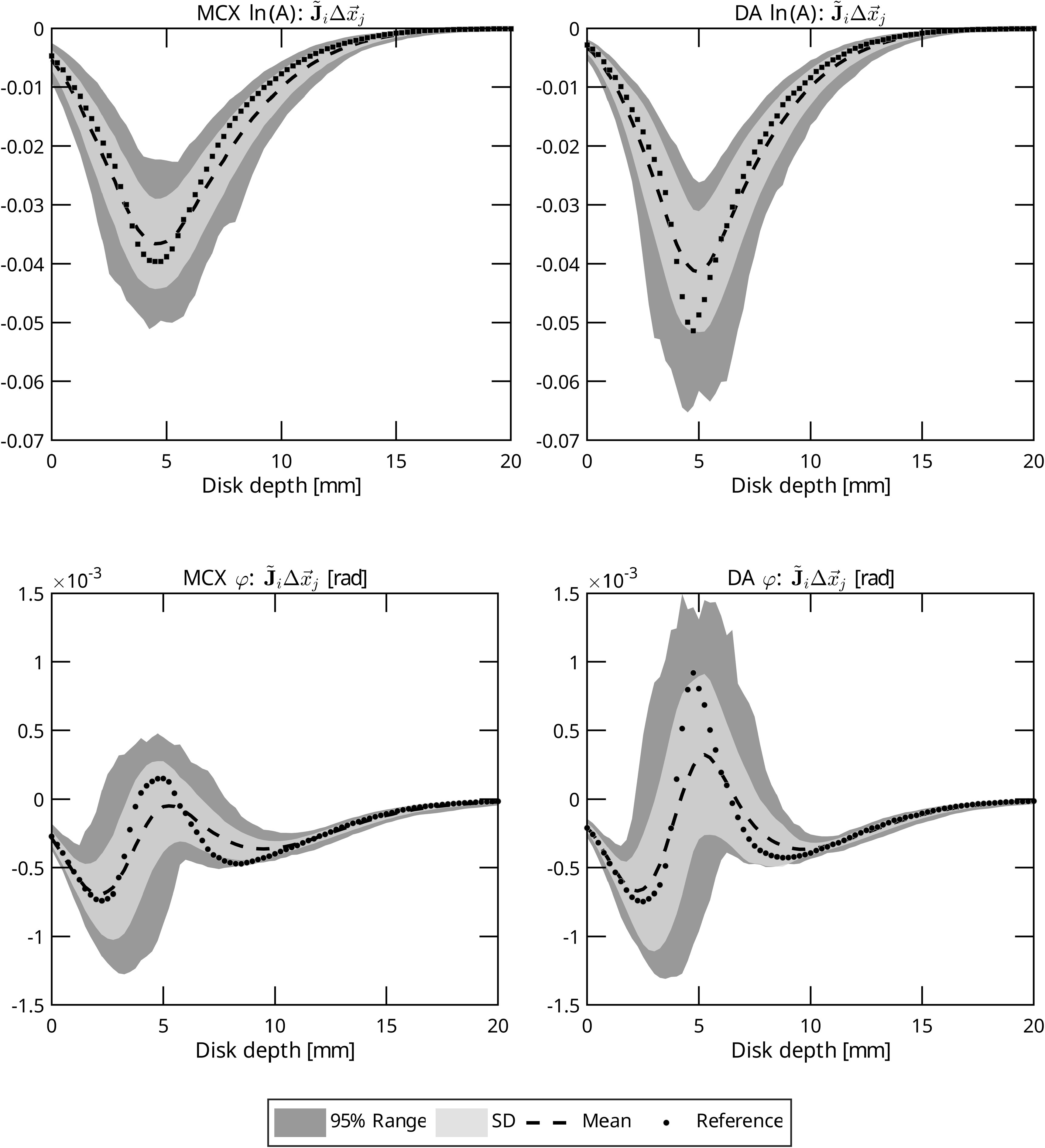
Derivative of the measurement with respect to the absorption coefficient in a slice of a cylinder drilled into the head at the midpoint between the source and the detector as a function of the depth of the slice for a source-detector pair at a distance 25 mm from each other. The derivative curve for the reference anatomy is compared to the mean derivative and the envelopes defined by the standard deviation (SD) and a range that covers 95% of the derivative values over the 165 anatomical atlas models. All four combinations of forward solvers (MCX and DA) and measurements (log-amplitude and phase) are considered.

\begin{eqnarray*}{\tilde{{\bf{J}}}}_{i}{\mathrm{\Delta }}{\vec{x}}_{j},\qquad i=1,...,N,\end{eqnarray*}

where ${\tilde{{\bf{J}}}}_{i}$ is the derivative of the considered measurement (i.e. its Jacobian as a row vector) with respect to the parametrization of the absorption coefficient in the *i*th anatomy, and the vector ${\mathrm{\Delta }}{\vec{x}}_{j}$ corresponds to a constant positive change in the absorption of the *j*th slice, i.e. it carries nonzero entries of magnitude 1 mm^−1^ only at the components corresponding to the absorption parametrization of the *j*th slice. Figure 8 visualizes such a derivative as a function of the depth of the perturbed slice for all combinations of the simulation methods and measurement types.

Figure 8 reveals a small discrepancy between the derivative in the reference anatomy and the mean of the derivatives over the whole atlas. However, much more significantly, the envelopes defined by the standard deviation and a range that covers 95% of the derivative values over the anatomical atlas vary significantly around the mean value of the derivative curve. Therefore the sensitivity of the considered measurement heavily depends on the anatomy. This effect is somewhat more pronounced for the DA compared to MCX.

## 5. Discussion

Model-based image reconstruction in DOT is prone to errors originating from inaccuracies in the optical head model. In this study, we focused on the manifestation of atlas-based modelling of the inner anatomy in the estimated transport of frequency-modulated light in newborns. We formed a sample of 165+1 late preterm to post-term neonate head models with the exact same exterior boundary and optode locations, but differing inner tissue boundaries, by registering 165 segmented models from the neonatal database by Collins-Jones *et al* (2021) to a reference subject’s exterior mask (Collins-Jones *et al* 2021). We simulated the FD measurements and their sensitivities to absorption changes, or Jacobians, for the reference and each individualized atlas model with the two most common forward solvers: MC simulations and solving the DA using FEM. The atlas approximation error in the absolute and difference measurements was compared to the change in the reference measurements following a tissue-wise or a local cerebral absorption perturbation, respectively.

For our set of anatomical models, we observed that using one of the 165 individualized atlas models instead of the reference subject’s own anatomy was on average a more significant source of error than limited mismodelling of the S&S (∼ +60 %) or GM (∼ −35 %) baseline absorption coefficient in the absolute measurements. The thinness of the S&S layer (minimum 2 mm), the lack of ‘short’ (2.15 mm; Brigadoi and Cooper 2015) SDS in our SDS range 7.5–55 mm and the log-amplitude scale contribute to the observed lower sensitivity to changes in the S&S baseline absorption. On the other hand, the difference in the reference model measurements due to a local spherical, physiologically reasonable 10% absorption increase (*R* = 5 mm, Δ*μ*_*a*_ = 5 · 10^−3^ mm^−1^) was clearly detectable over the error in the linear estimates for the difference computed with the registered Jacobians. This is in line with the pre-assumption that some modelling errors are effectively canceled out in DOT difference imaging (Bluestone *et al* 2001, Nissilä *et al* 2005b). The observation also gives reason to believe that individual atlas models could potentially be used with some accuracy over a gestational age range of several weeks in difference imaging. For estimating the tissue-specific baseline optical parameters by fitting simulated to real absolute measurements as in (Maria *et al* 2022), less (age-related) inaccuracy in the segmented anatomical model is preferable in neonates, though averaging over multiple subjects can improve the accuracy.

The rapid convergence of the eigenvalues in figure 7 for the covariance matrix of the approximation error in the difference measurements suggests that the approximation error method could be implemented to account for the error in the inverse problem of image reconstruction. However, applying the method to the linearized reconstruction problem is not automatic based on previously published research as the approximation error manifests itself in the Jacobian and, moreover, the applicability of the method depends on the fine interplay between the approximation error and the strength of the signal of interest. For absolute measurements, the implementation of the approximation error method would be more straightforward, but the overlap of the essential eigenvalues and projections in figure 4 is not optimal for the approach.

It is important to consider the observed magnitude of the approximation error, especially in the absolute measurements, in the correct context: The original motivation given by Collins-Jones *et al* (2021) for collecting the neonatal database was providing individual atlas models for a dense, ±1 week age range, since brain anatomies do not match well over the different gestational ages due to the rapid brain growth and maturation. In this study, the age range of included neonates was a relatively large 34.0–44.3 weeks of combined gestational and chronological age. Thus, our models arguably contain samples of relatively large segmentation variation, which is further highlighted by the thinness of the S&S and the proximity of the brain to the surface in neonates. The registration process can also amplify the variation in some regions, and especially the width of the S&S layer changes during the exact surface match. The variation in the median S&S thickness under the cranial 10–5 points is already quite large in the original models, for example, figure 8 in Collins-Jones *et al* (2021) has range 2.5–6.5 mm at PMA of 40 weeks. The variation could relate partly to the short-duration cranial deformations that can occur in newborns. Segmenting the S&S layer from MR images can also be challenging (Collins-Jones *et al* 2021).

The presented registration procedure was modified from our recent model generation pipeline (Hirvi 2019, Maria *et al* 2022), e.g. by adding the exact matching of the atlas to the reference exterior surface to eliminate variation in the simulated measurements due to the head geometry or the positioning of the optodes. However, typically registration is based on cranial landmarks instead of matching the exact shape of the reference geometry (Wu *et al* 2014, 2015, Maria *et al* 2022). Thus, in practice there is always also some error in the locations of the optodes due to both the differences in the head geometry and the independent measurement error in the locations, which are not accounted for.

A limitation of this study is that the results are to some extent dependent on the selected reference neonate. However, the choice can be considered reasonable as the measurements simulated in the reference model are close to the mean simulated values over all of the registered subjects. Another limitation is that we have considered log-amplitude and phase shift measurements separately, whereas in real FD DOT, both data types would be utilized simultaneously, and despite some correlation, the combined outcome is likely to be better than either individually. A third limitation is that the study only considers variation in the segmentation of five homogeneous tissue types, which does not fully describe the highly heterogeneous head. A fourth limitation is that the two-compartment CSF model was only implemented in the MC solver due to numerical issues with the FEM, fundamentally arising from the approximations made in the DA. For MC, we observed that separating the sulci as clearer regions from the subarachnoid increased the variation in the absolute measurements simulated in the different atlases with individual sulci locations. Finally, the voxel-based models in MC represent the exterior tissue boundaries with less accuracy than the mesh models in the DA, leading to differences particularly in reflections at the boundary. The results in this study do not show major differences between MC and DA, but in the future, once the computation of the FD Jacobians is implemented to the mesh-based (MMC; Fang 2010) or mesh-voxel hybrid (SVMC; Yan and Fang 2020) MC domain models, their usage may improve imaging accuracy especially if shorter SDS are included.

The natural follow-up for this study is to observe how the actual reconstructions of simulated perturbations in the reference head change when the individual atlas-based Jacobians are used instead of the reference Jacobian. This would enable estimating the level of spatial and contrast-wise accuracy that can be reached with the atlas-based reconstructions. Then we could potentially suggest a reasonable age range for individual neonatal atlas models in DOT difference imaging.

In the future, the presented CSF segmentation algorithm could turn out as a valuable tool also in other model-based imaging modalities, though the optical properties of CSF still require further research, which could also consider the highly-absorbing blood vessels. In addition, our registration approach could be used to build a probabilistic neonatal atlas model, which might perform better than an individual or population-level atlas with fixed segmentation (Heiskala *et al* 2009b).

## 6. Conclusion

In this work, we considered using late preterm to post-term individual-level atlas models for a term reference neonate in frequency-domain (FD) diffuse optical tomography (DOT). We first developed a quick automatic segmentation algorithm for separating the semidiffusive subarachnoid cerebrospinal fluid (CSF) layer from the clearer CSF in the brain sulci and ventricles. Then we individualized 165 segmented voxel- and mesh-based newborn head models to have the exact same exterior shape and high-density optode locations, but differing inner anatomy, to the reference neonate. We programmed an in-house finite element -solver for the diffusion approximation of light transport, and implemented the computation of the FD sensitivity profiles to the Monte Carlo eXtreme (MCX) photon simulation software. We observed that for more accurate estimation of the absolute measurements, using the subject’s own segmented anatomical model is preferable due to the large inter-individual variation over the considered wide range of gestational ages. The atlas models could potentially be used in DOT difference imaging, and the expected accuracy as well as the possibility of enhancing the image reconstruction with the approximation error method are of interest in further study.

## Data Availability

The data that support the findings of this study are available upon request from the authors.

## Funding

The Vilho, Yrjö and Kalle Väisälä Foundation of the Finnish Academy of Science and Letters (to PH; grant numbers 190017 and 200015). Academy of Finland (to AH, NH, PH and TK; decisions 336788 and 336789). Päivikki and Sakari Sohlberg Foundation (to IN). US National Institute of Health (NIH) award R01-GM114365 (to QF). The UCL neonatal models were obtained using data made available from the Developing Human Connectome Project funded by the European Research Council under the European Union’s Seventh Framework Programme (FP/2007-2013)/ERC Grant Agreement no. (319456).

## Disclosures

The authors declare no conflicts of interest.

## References

[pmbacd48cbib1] Arridge S R (1999). Optical tomography in medical imaging. Inverse Problems.

[pmbacd48cbib2] Arridge S R, Hebden J C (1997). Optical imaging in medicine: II. Modelling and
reconstruction. Phys. Med. Biol..

[pmbacd48cbib3] Arridge S R, Kaipio J P, Kolehmainen V, Schweiger M, Somersalo E, Tarvainen T, Vauhkonen M (2006). Approximation errors and model reduction with an application in
optical diffusion tomography. Inverse Problems.

[pmbacd48cbib4] Arridge S R, Lionheart W R B (1998). Nonuniqueness in diffusion-based optical tomography. Opt. Lett..

[pmbacd48cbib5] Arridge S R, Schweiger M (1995). Photon-measurement density functions. Part 2: Finite-element-method
calculations. Appl. Opt..

[pmbacd48cbib6] Benko N, Luke E, Alsanea Y, Coats B (2020). Spatial distribution of human arachnoid trabeculae. J. Anat..

[pmbacd48cbib7] Binzoni T, Sassaroli A, Torricelli A, Spinelli L, Farina A, Durduran T, Cavalieri S, Pifferi A, Martelli F (2017). Depth sensitivity of frequency domain optical measurements in
diffusive media. Biomed. Opt. Express.

[pmbacd48cbib8] Bluestone A Y, Abdoulaev G, Schmitz C H, Barbour R L, Hielscher A H (2001). Three-dimensional optical tomography of hemodynamics in the human
head. Opt. Express.

[pmbacd48cbib9] Boas D A, Culver J P, Stott J J, Dunn A K (2002). Three dimensional Monte Carlo code for photon migration through
complex heterogeneous media including the adult human head. Opt. Express.

[pmbacd48cbib10] Brigadoi S, Aljabar P, Kuklisova-Murgasova M, Arridge S R, Cooper R J (2014). A 4D neonatal head model for diffuse optical imaging of pre-term to
term infants. NeuroImage.

[pmbacd48cbib11] Brigadoi S, Cooper R J (2015). How short is short? Optimum source-detector distance for
short-separation channels in functional near-infrared spectroscopy. Neurophotonics.

[pmbacd48cbib12] Candiani V, Hyvönen N, Kaipio J P, Kolehmainen V (2021). Approximation error method for imaging the human head by electrical
impedance tomography. Inverse Problems.

[pmbacd48cbib13] Collins-Jones L H, Arichi T, Poppe T, Billing A, Xiao J, Fabrizi L, Brigadoi S, Hebden J C, Elwell C E, Cooper R J (2021). Construction and validation of a database of head models for
functional imaging of the neonatal brain. Hum. Brain Mapp..

[pmbacd48cbib14] Cooper R J, Caffini M, Dubb J, Fang Q, Custo A, Tsuzuki D, Fischl B, Wells W, Dan I, Boas D A (2012). Validating atlas-guided DOT: A comparison of diffuse optical
tomography informed by atlas and subject-specific anatomies. NeuroImage.

[pmbacd48cbib15] Custo A, Boas D A, Tsuzuki D, Dan I, Mesquita R, Fischl B, Grimson W E L, Wells W (2010). Anatomical atlas-guided diffuse optical tomography of brain
activation. NeuroImage.

[pmbacd48cbib16] Custo A, Wells W M, Barnett A H, Hillman E M C, Boas D A (2006). Effective scattering coefficient of the cerebral spinal fluid in adult
head models for diffuse optical imaging. Appl. Opt..

[pmbacd48cbib17] Dehghani H, Arridge S R, Schweiger M, Delpy D T (2000). Optical tomography in the presence of void regions. J. Opt. Soc. Am. A.

[pmbacd48cbib18] Doulgerakis M, Eggebrecht A T, Dehghani H (2019). High-density functional diffuse optical tomography based on
frequency-domain measurements improves image quality and spatial
resolution. Neurophotonics.

[pmbacd48cbib19] Fang Q (2010). Mesh-based Monte Carlo method using fast ray-tracing in Plücker
coordinates. Biomed. Opt. Express.

[pmbacd48cbib20] Fang Q, Boas D A (2009a). Tetrahedral mesh generation from volumetric binary and grayscale
images,.

[pmbacd48cbib21] Fang Q, Boas D A (2009b). Monte Carlo simulation of photon migration in 3D turbid media
accelerated by graphics processing units. Opt. Express.

[pmbacd48cbib22] Fang Q, Yan S (2022). MCX cloud–a modern, scalable, high-performance and in-browser Monte
Carlo simulation platform with cloud computing. J. Biomed. Opt..

[pmbacd48cbib23] Farina A (2015). *In-vivo* multilaboratory investigation of the optical
properties of the human head. Biomed. Opt. Express.

[pmbacd48cbib24] Ferradal S L, Eggebrecht A T, Hassanpour M, Snyder A Z, Culver J P (2014). Atlas-based head modeling and spatial normalization for high-density
diffuse optical tomography: *In vivo* validation
against fMRI. NeuroImage.

[pmbacd48cbib25] Fukui Y, Ajichi Y, Okada E (2003). Monte Carlo prediction of near-infrared light propagation in realistic
adult and neonatal head models. Appl. Opt..

[pmbacd48cbib26] Garel C, Chantrel E, Brisse H, Elmaleh M, Luton D, Oury J-F, Sebag G, Hassan M (2001). Fetal cerebral cortex: Normal gestational landmarks identified using
prenatal MR imaging. Am. J. Neuroradiol..

[pmbacd48cbib27] Gibson A P, Hebden J C, Arridge S R (2005). Recent advances in diffuse optical imaging. Phys. Med. Biol..

[pmbacd48cbib28] Gustafsson T, McBain G D (2020). scikit-fem: A Python package for finite element
assembly. J. Open Source Software.

[pmbacd48cbib29] Hannukainen A, Harhanen L, Hyvönen N, Majander H (2015). Edge-promoting reconstruction of absorption and diffusivity in optical
tomography. Inverse Problems.

[pmbacd48cbib30] Hartmann K, Stein K-P, Neyazi B, Sandalcioglu I E (2019). First *in vivo* visualization of the human
subarachnoid space and brain cortex via optical coherence
tomography. Ther. Adv. Neurol. Disord..

[pmbacd48cbib31] Haskell R C, Svaasand L O, Tsay T-T, Feng T-C, McAdams M S, Tromberg B J (1994). Boundary conditions for the diffusion equation in radiative
transfer. J. Opt. Soc. Am. A.

[pmbacd48cbib32] Heino J, Somersalo E (2002). Estimation of optical absorption in anisotropic
background. Inverse Problems.

[pmbacd48cbib33] Heiskala J (2009). Accurate modelling of tissue properties in diffuse optical imaging of
the human brain. PhD thesis.

[pmbacd48cbib34] Heiskala J, Hiltunen P, Nissilä I (2009a). Significance of background optical properties, time-resolved
information and optode arrangement in diffuse optical imaging of term
neonates. Phys. Med. Biol..

[pmbacd48cbib35] Heiskala J, Kolehmainen V, Tarvainen T, Kaipio J P, Arridge S R (2012). Approximation error method can reduce artifacts due to scalp blood
flow in optical brain activation imaging. J. Biomed. Opt..

[pmbacd48cbib36] Heiskala J, Kotilahti K, Lipiäinen L, Hiltunen P, Grant P E, Nissilä I, Pogue B W, Cubeddu R (2007). Optical tomographic imaging of activation of the infant auditory
cortex using perturbation Monte Carlo with anatomical a priori
information.

[pmbacd48cbib37] Heiskala J, Pollari M, Metsäranta M, Grant P E, Nissilä I (2009b). Probabilistic atlas can improve reconstruction from optical imaging of
the neonatal brain. Opt. Express.

[pmbacd48cbib38] Hillman E M C, Devor A, Bouchard M B, Dunn A K, Krauss G W, Skoch J, Bacskai B J, Dale A M, Boas D A (2007). Depth-resolved optical imaging and microscopy of vascular compartment
dynamics during somatosensory stimulation. NeuroImage.

[pmbacd48cbib39] Hintz S R, Cheong W-F, Van Houten J P, Stevenson D K, Benaron D A (1999). Bedside imaging of intracranial hemorrhage in the neonate using light:
Comparison with ultrasound, computed tomography, and magnetic resonance
imaging. Pediatric Res..

[pmbacd48cbib40] Hirvi P (2019). Generating head models for diffuse optical tomography of the child
brain. Master’s thesis.

[pmbacd48cbib41] Hyvönen N (2002). Analysis of optical tomography with non-scattering
regions. Proc. of the Edinburgh Mathematical Society.

[pmbacd48cbib42] Jönsson E H (2018). Affective and non-affective touch evoke differential brain responses
in 2-month-old infants. NeuroImage.

[pmbacd48cbib43] Kaipio J, Somersalo E (2005). Statistical and Computational Inverse Problems, volume 160 of Applied
Mathematical Sciences.

[pmbacd48cbib44] Koikkalainen J, Lötjönen J (2004). Reconstruction of 3D head geometry from digitized point sets: An
evaluation study. IEEE Trans. Inf. Technol. Biomed..

[pmbacd48cbib45] Kolehmainen V, Schweiger M, Nissilä I, Tarvainen T, Arridge S R, Kaipio J P (2009). Approximation errors and model reduction in three-dimensional diffuse
optical tomography. J. Opt. Soc. Am. A.

[pmbacd48cbib46] Makropoulos A (2018). The developing human connectome project: A minimal processing pipeline
for neonatal cortical surface reconstruction. NeuroImage.

[pmbacd48cbib47] Maria A, Hirvi P, Kotilahti K, Heiskala J, Tuulari J J, Karlsson L, Karlsson H, Nissilä I (2022). Imaging affective and non-affective touch processing in two-year-old
children. NeuroImage.

[pmbacd48cbib48] Maria A, Nissilä I, Shekhar S, Kotilahti K, Tuulari J J, Hirvi P, Huotilainen M, Heiskala J, Karlsson L, Karlsson H (2020). Relationship between maternal pregnancy-related anxiety and infant
brain responses to emotional speech–a pilot study. J. Affective Disorders.

[pmbacd48cbib49] Mortazavi M M (2018). Subarachnoid trabeculae: A comprehensive review of their embryology,
histology, morphology, and surgical significance. World Neurosurgery.

[pmbacd48cbib50] Mozumder M, Tarvainen T, Arridge S R, Kaipio J, Kolehmainen V (2013). Compensation of optode sensitivity and position errors in diffuse
optical tomography using the approximation error approach. Biomed. Opt. Express.

[pmbacd48cbib51] Mozumder M, Tarvainen T, Kaipio J P, Arridge S R, Kolehmainen V (2014). Compensation of modeling errors due to unknown domain boundary in
diffuse optical tomography. J. Opt. Soc. Am. A.

[pmbacd48cbib52] Nissilä I, Noponen T, Heino J, Kajava T, Katila T, Lin J C (2005b). Diffuse optical imaging. Advances in Electromagnetic Fields in Living Systems.

[pmbacd48cbib53] Nissilä I, Noponen T, Kotilahti K, Katila T (2005a). Instrumentation and calibration methods for the multichannel
measurement of phase and amplitude in optical tomography. Rev. Sci. Instrum..

[pmbacd48cbib54] Okada E, Delpy D T (2003). Near-infrared light propagation in an adult head model. I. Modeling of
low-level scattering in the cerebrospinal fluid layer. Appl. Opt..

[pmbacd48cbib55] Okada E, Firbank M, Schweiger M, Arridge S R, Cope M, Delpy D T (1997). Theoretical and experimental investigation of near-infrared light
propagation in a model of the adult head. Appl. Opt..

[pmbacd48cbib56] Sassaroli A, Martelli F (2012). Equivalence of four Monte Carlo methods for photon migration in turbid
media. J. Opt. Soc. Am. A.

[pmbacd48cbib57] Schweiger M, Arridge S R, Hiraoka M, Delpy D T (1995). The finite element method for the propagation of light in scattering
media: Boundary and source conditions. Med. Phys..

[pmbacd48cbib58] Shekhar S, Maria A, Kotilahti K, Huotilainen M, Heiskala J, Tuulari J J, Hirvi P, Karlsson L, Karlsson H, Nissilä I (2019). Hemodynamic responses to emotional speech in two-month-old infants
imaged using diffuse optical tomography. Sci. Rep..

[pmbacd48cbib59] Tran A P, Yan S, Fang Q (2020). Improving model-based functional near-infrared spectroscopy analysis
using mesh-based anatomical and light-transport models. Neurophotonics.

[pmbacd48cbib60] Wang L, Jacques S L, Zheng L (1995). MCML—Monte Carlo modeling of light transport in multi-layered
tissues. Comput. Methods Programs Biomed..

[pmbacd48cbib61] Wu X, Eggebrecht A T, Ferradal S L, Culver J P, Dehghani H (2014). Quantitative evaluation of atlas-based high-density diffuse optical
tomography for imaging of the human visual cortex. Biomed. Opt. Express.

[pmbacd48cbib62] Wu X, Eggebrecht A T, Ferradal S L, Culver J P, Dehghani H (2015). Evaluation of rigid registration methods for whole head imaging in
diffuse optical tomography. Neurophotonics.

[pmbacd48cbib63] Yan S, Fang Q (2020). Hybrid mesh and voxel based Monte Carlo algorithm for accurate and
efficient photon transport modeling in complex bio-tissues. Biomed. Opt. Express.

[pmbacd48cbib64] Yao R, Intes X, Fang Q (2018). Direct approach to compute Jacobians for diffuse optical tomography
using perturbation Monte Carlo-based photon ‘replay’. Biomed. Opt. Express.

